# Genome-wide identification of Hami melon miRNAs with putative roles during fruit development

**DOI:** 10.1371/journal.pone.0180600

**Published:** 2017-07-24

**Authors:** Hong Zhang, Lan Yin, Huaisong Wang, Guangzhi Wang, Xinli Ma, Meihua Li, Haibo Wu, Qiushi Fu, Yi Zhang, Hongping Yi

**Affiliations:** 1 Hami Melon Research Center, Xinjiang Academy of Agricultural Sciences, Urumqi, Xinjiang, China; 2 ABLife, Inc., Wuhan, Hubei, China; 3 Institute of Vegetables and Flowers, Chinese Academy of Agricultural Sciences, Beijing, China; Dokuz Eylul Universitesi, TURKEY

## Abstract

MicroRNAs represent a family of small endogenous, non-coding RNAs that play critical regulatory roles in plant growth, development, and environmental stress responses. Hami melon is famous for its attractive flavor and excellent nutritional value, however, the mechanisms underlying the fruit development and ripening remains largely unknown. Here, we performed small RNA sequencing to investigate the roles of miRNAs during Hami melon fruit development. Two batches of flesh samples were collected at four fruit development stages. Small RNA sequencing yielded a total of 54,553,424 raw reads from eight libraries. 113 conserved miRNAs belonging to 30 miRNA families and nine novel miRNAs comprising nine miRNA families were identified. The expression of 42 conserved miRNAs and three Hami melon-specific miRNAs significantly changed during fruit development. Furthermore, 484 and 124 melon genes were predicted as putative targets of 29 conserved and nine Hami melon-specific miRNA families, respectively. GO enrichment analysis were performed on target genes, “transcription, DNA-dependent”, “rRNA processing”, “oxidation reduction”, “signal transduction”, “regulation of transcription, DNA-dependent”, and “metabolic process” were the over-represented biological process terms. Cleavage sites of six target genes were validated using 5’ RACE. Our results present a comprehensive set of identification and characterization of Hami melon fruit miRNAs and their potential targets, which provide valuable basis towards understanding the regulatory mechanisms in programmed process of normal Hami fruit development and ripening. Specific miRNAs could be selected for further research and applications in breeding practices.

## Introduction

MicroRNAs are a class of endogenous noncoding small RNAs, 20–24 nucleotides (nt), which regulate gene expression at post-transcriptional levels via endonucleolytic cleavage or translational inhibition [1]. In plants, the primary miRNA transcripts are first transcribed by RNA polymerase II and processed by Dicer-like 1 to generate stem-loop miRNA:miRNA* duplex [2]. Then, the duplex disassociates and mature miRNAs are incorporated to the RNA-induced silencing complex. Argonaute proteins are a core component of this complex, which could bind to the small RNAs and enzymatically cleave the complementary mRNAs or inhibit its translation [1, 3].

Numerous evidences have pointed that miRNAs play vital roles in a myriad of biological processes in plants, such as growth and development, as well as responses to environmental stresses [4–6]. For instance, in both Arabidopsis and maize, miR156 and miR172 act antagonistically in developmental transitions through the regulation of their targets squamosa promoter binding protein-like (SPL) and apetala 2 (AP2) transcription factors, respectively [4, 7, 8]. miR160 is demonstrated to target auxin response factors (ARFs) to function in Arabidopsis root development [9, 10]. The expression of miR164 gradually declines with aging, leading to the enhancement of a NAM, ATAF, and CUC (NAC) transcription factor, which positively regulates aging-induced cell death in Arabidopsis leaves [11]. miR319 modulates the activity of TEOSINTE BRANCHED 1, cycloidea and PCF (TCP) transcription factors by controlling the fate of leaves and flowers growth, and its overexpression could cause plants to stay green much longer [4, 12]. Both miR159a and miR159b were verified to interact with an MYB transcription factor during the course of strawberry receptacle development [13]. miRNAs also play important roles in fleshy fruit development. miR156-SPL module is involved in meristem maintenance in the developing ovaries of tomato, controlling initial steps of fleshy fruit development and determinacy [14]. miR396 negatively regulates the fruit size and weight of tomato fruit [15]. In recent years, high-throughput sequencing has been used for the identification and expression profiling of miRNAs in many fruit crops, such as tomato [16–19], melon [20], apple [21], grape [22], strawberry [23], papaya [24], sweet orange [25], and pear [26]. As an established model system, the molecular mechanisms of tomato fruit development and ripening have been investigated through high-throughput sequencing. Many fruit development and ripening associated miRNAs were identified from tomato young green fruits and, lately, from several fruit developmental stages [17–19, 27]. miR156 and miR172 are identified to actively modulate the known ripening regulators, *COLORLESS NON-RIPENING (CNR)* and *AP2a*, respectively during tomato fruit ripening [19]. miR393 is identified to be involved in the initiation of fruit development by targeting TRANSPORT INHIBITOR RESPONSE 1 (TIR1) and its homologues [19].

Hami melon is an important horticultural fruit cultivated in the Xinjiang Uyghur Autonomous Region of China. It is popular with customers because of its large size, sugar content, fragrance, good taste and crispy flesh texture [28, 29]. Using Roche 454 pyrosequencing platform, Gonzalez-Ibeas et al [20] have previously identified conserved and melon-specific miRNAs from multiple melon tissues, including fruits, ovaries and cotyledons, and they investigated the roles of miRNAs in fruit development by comparing the differential expression profiles at two stages. With the genome of melon being decoded [30], the molecular research on melon is greatly accelerated. A close examination of the dynamic regulation of miRNAs during sequential stages of Hami melon fruit development would help us understand the molecular mechanisms of fruit development in Hami melon.

In the present study, the miRNA expression profiles of Hami melon at four fruit developmental stages were analyzed. We have obtained a total of 54,553,424 raw reads from the fruit, which allowed us to identify 113 conserved miRNAs belonging to 30 miRNA families and nine novel miRNAs comprising nine miRNA families. Furthermore, the potential target genes of miRNAs were predicted based on previously established rules in plants. This study provided a valuable resource to examine how global genome expression are regulated by miRNAs during melon fruit development. Specific miRNAs may be applied in breeding crops with improved properties to fulfill various types of demand in the market.

## Results

### Small RNA sequence (sRNA-seq) and data analysis

To investigate the miRNAs regulatory network during Hami melon fruit development, eight small RNA (sRNA) libraries were constructed from the fruit flesh of Hami melon collected at 10 days after flowering (10DAF), 20 days after flowering (20DAF), 30 days after flowering (30DAF) and 40 days after flowering (40DAF), respectively. High-throughput sequencing generated 4,615,916 to 8,262,912 raw sRNA readouts (Table 1). After discarding adaptor contaminants, low-quality sequences, and reads with length shorter than 18 nt and greater than 30 nt, we obtained the reliable clean reads ranging from 3,766,406 to 6,379,19. We compared our sRNA reads data with the melon high-throughput sRNA data published before [20], and found that the majority of sRNAs in the previous high-throughput study were only sequenced a few times, while in our database nearly half of sRNAs in each library were sequenced over 30 times (S1 Fig). Therefore, our database could better quantify the expression levels of melon fruit sRNAs than the published melon sRNA data. We merged the clean reads from the first batch and second batch, the sRNA singleton rate of each developmental stage was calculated. As shown in Table 1, the average singleton rate of Hami melon was 62%, which is similar with those of Arabidopsis (65%), rice (82%), poplar (73%), *Cunninghamia lanceolata* (74%) [31], and *Salicornia europaea* (77%) [32].

**Table 1:** Summary of sRNA-seq data from the eight small RNA libraries.

Category	Developmental stages							
	10DAF_2013	10DAF_2014	20DAF_2013	20DAF_2014	30DAF_2013	30DAF_2014	40DAF_2013	40DAF_2014
Raw reads	7,709,000 (100)	7,006,530 (100)	6,838,455 (100)	8,262,912 (100)	7,180,516 (100)	4,615,916 (100)	5,729,417 (100)	7,210,678 (100)
Clean reads (18–30 nt)	4,703,776 (61.02)	5,303,174 (75.69%)	5,218,976 (76.32)	6,379,195 (77.20)	5,692,948 (79.28)	3,766,406 (81.60)	3,800,695 (66.34)	5,443,842 (75.50)
Unique sequence reads	2,766,920 (35.89)	1,837,258 (26.22)	3,215,074 (47.01)	2,031,994 (24.59)	3,441,573 (47.93)	1,191,234 (25.81)	2,297,275 (40.10)	1,782,240 (24.72)
Total unique sequence reads	4,396,588		4,992,103		4,450,165		3,843,316	
Singleton sequence reads	2,415,186 (31.33)	386,401 (5.51)	2,773,387 (40.56)	453,908 (5.49)	2,942,608 (40.98)	263,699 (5.71)	1,971,905 (34.42)	417,531 (5.79)
Total singleton sequence reads	2,659,612		3,051,684		3,103,150		2,246,222	

DAF represents days after flowering. 2013 represents the first batch samples collected in 2013. 2014 represents the second batch samples collected in 2014.

Read counts for each sample are expressed in numbers (left) or as a percentage of the raw reads (right). The number of total unique sequence reads and total singleton sequence reads at each developmental stage was calculated after the sRNA data from the first and second batch were merged together.

The high-quality clean reads were searched against Rfam database (version 12.0), the known non-coding RNAs, including lncRNAs, miRNAs, rRNAs, tRNAs, and other families, were annotated (Table 2). sRNA sequences annotated as rRNAs and tRNAs were removed. The remaining sequences were used for the alignment against the known plant miRNAs in miRBase database (v21, July 2014) [33].

**Table 2:** Rfam annotation of clean reads obtained after sequence data processing.

Class	Developmental stages							
	10DAF_2013	10DAF_2014	20DAF_2013	20DAF_2014	30DAF_2013	30DAF_2014	40DAF_2013	40DAF_2014
Clean reads	4,703,776 (100)	5,303,174 (100)	5,218,976 (100)	6,379,195 (100)	5,692,948 (100)	3,766,406 (100)	3,800,695 (100)	5,443,842 (100)
Total match	736,076 (15.65)	848,899 (16.01)	621,513 (11.91)	1,261,168 (19.77)	680,071 (11.95)	252,725 (6.71)	399,644 (10.52)	422,535 (7.76)
lncRNA	1 (0.00)	0 (0.00)	2 (0.00)	1 (0.00)	1 (0.00)	3 (0.00)	1 (0.00)	0 (0.00)
miRNA	4,234 (0.09)	8,528 (0.16)	17,457(0.33)	24,241 (0.38)	12,967 (0.23)	19,972 (0.53)	4,867 (0.13)	10,365 (0.19)
rRNA	209,594 (4.46)	123,807 (2.33)	151,131 (2.90)	166,888 (2.62)	162,908 (2.86)	63,290 (1.68)	91,758 (2.41)	162,042 (2.98)
snRNA	0 (0.00)	0 (0.00)	0 (0.00)	0 (0.00)	0 (0.00)	0 (0.00)	0 (0.00)	0 (0.00)
tRNA	152,914 (3.25)	65,560 (1.24)	37,054 (0.71)	31,236 (0.49)	36,080 (0.63)	15,808 (0.42)	37,185 (0.98)	29,130 (0.54)
Others	369,333 (7.85)	651,004 (12.28)	415,869 (7.97)	1,038,802 (16.28)	468,115 (8.22)	153,652 (4.08)	265,833 (6.99)	220,998 (4.06)

DAF represents days after flowering. 2013 represents the first batch samples collected in 2013. 2014 represents the second batch samples collected in 2014.

Read counts for each sample are expressed in numbers (left) or as a percentage of the input clean reads (right).

**Fig 1:**
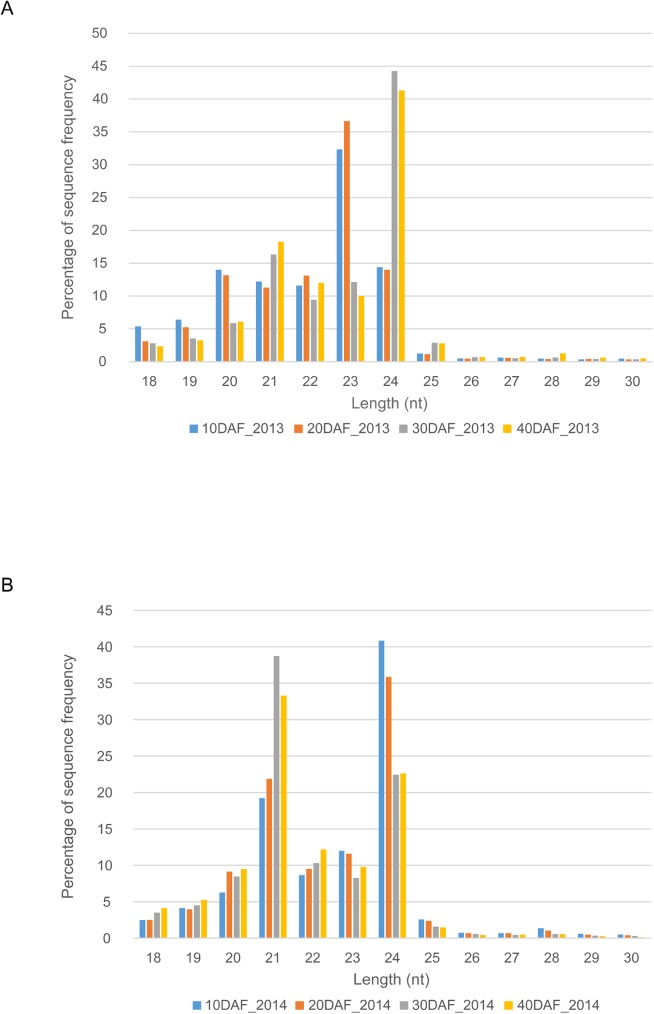
**Size distribution of small RNA sequences in 10DAF, 20DAF, 30DAF and 40DAF libraries from the first batch (A) and second batch (B).** nt, nucleotides. DAF represents days after flowering. 2013 represents the first batch samples collected in 2013. 2014 represents the second batch samples collected in 2014.

The distribution of processed sequences with different lengths in the small RNA datasets was summarized in Fig 1. The majority of sRNAs were in the range of 20–24 nt in length, which are the typical size range generated by Dicer [34, 35]. In the first batch, 23 nt sRNAs were the most abundant in 10DAF and 20DAF libraries, and 24nt for 30DAF and 40DAF libraries; while in the second batch, 24 nt was the major size of sRNAs in 10DAF and 20DAF libraries, and 21 nt was the major size in 30DAF and 40DAF libraries. Endogenous sRNAs of 24 nt are the most abundant sRNAs in some fruit crops, such as cucumber [36], apple [21], sweet orange [25], pear [26] and aphid susceptible and resistant melon breeding lines [37]. In contrast, 21 nt is the predominant size of sRNAs identified from RNA viruses susceptible and resistant melon cultivars [20]. Among the conserved miRNAs identified in Hami melon, 73.53% were 21 nt, 16.18% were 20 nt, only a small fraction (1 to 3%) represented by 22, 23, and 24 nt. To clarify the difference of the length distribution of sRNA sequences between the two separate batches, we analyzed the length distribution pattern of each annotated sRNA family (Table 2). Considering that only a small part of the non-coding sequences were annotated as lncRNAs (Table 2), the annotated lncRNAs were excluded from this analysis. As shown in S2 Fig, miRNAs, rRNAs, tRNAs, and other families all showed different length distribution patterns between the first and second batches, they may partly contribute to the difference between the two separate batches exhibited in Fig 1. Notably, the unannotated sRNAs showed the most similar length distribution pattern with the total sRNAs (18–30 nt). Since the unannotated sRNAs represented the major part of the total sRNAs (18–30 nt) (Table 2), the unannotated sRNAs should account for the difference of the length distribution of sRNA sequences between the two separate batches. Besides, we also plotted the length distribution of the miRNAs identified by aligning to known miRNAs in miRBase or computational methods (S3 Fig). The length distribution pattern of miRNAs from the first batch dataset showed similar trends to that of miRNAs from the second batch dataset.

### Identification of conserved miRNAs in Hami melon

In order to identify the conserved miRNAs in Hami melon fruit, high-quality unique sequences from the eight libraries were matched to the repository of plant miRNAs in miRBase using Bowtie2. One mismatch was allowed in Bowtie2 alignment that resulted in the identification of 113 conserved miRNAs belonging to 30 families in Hami melon (S1 Table). The conserved miRNA were 20–24 nt in length with 21 nt and 20 nt as the major size classes. The total counts of conserved miRNAs were higher in 20DAF and 30DAF libraries when compared with the other libraries, suggesting conserved miRNAs fast accumulated at the stages of 20DAF and 30DAF. The precursor sequences of Hami melon conserved miRNAs were listed in S2 Table.

### Identification of novel Hami melon-specific miRNAs

Unique sequences that did not match with conserved plant miRNAs were matched to MELONOMICS genome sequence (v3.5) [38] using miRDeep algorithm [39]. The non-coding potential precursors were manually inspected with Mfold [40] and the minimal folding free energy index (MFEI) for each sequence was calculated [41]. Finally, nine novel miRNAs belonging to nine families were identified in Hami melon (S3 Table). Most of the novel miRNAs were 21 or 24 nt, only cme-miR8 was 22 nt. The length of miRNA precursors specific to Hami melon varied within the range of 68–101 nt. The MFEIs varied between 0.55 and 1.29 with an average of 0.99, which were in agreement with the parameters revealed in other plant miRNAs [41]. The total counts of Hami melon-specific miRNAs was highest in 10DAF library compared with other libraries, and were reduced during the fruit development. The secondary structures predicted for each novel miRNAs were shown in S4 Fig.

### Expression profiles of conserved and novel miRNAs during Hami melon development

**Fig 2:**
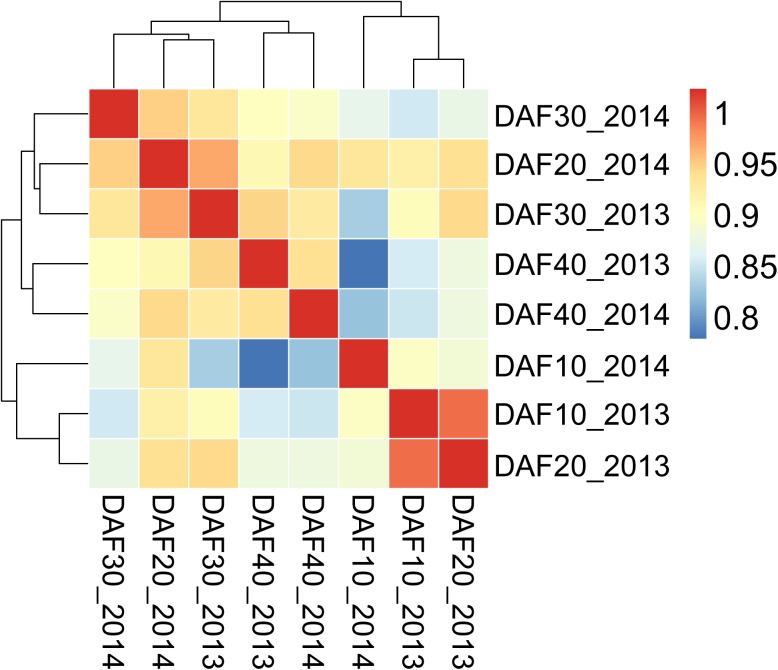
Hierarchical clustering of the expression profiles of all expressed miRNAs during Hami melon fruit development. DAF represents days after flowering. 2013 represents the first batch samples collected in 2013. 2014 represents the second batch samples collected in 2014.

Each batch was considered as a biological replicate set, and all miRNA counts were normalized to transcripts per million (TPM). The heat map showed that the expression patterns of identified miRNAs at 10DAF, 30DAF and 40DAF were distinct from each other (Fig 2). While the samples from 20DAF libraries could not form a cluster, the first batch sample of 20DAF was clustered with 10DAF samples, and the secondary batch sample of 20DAF was clustered with 30DAF samples.

**Fig 3:**
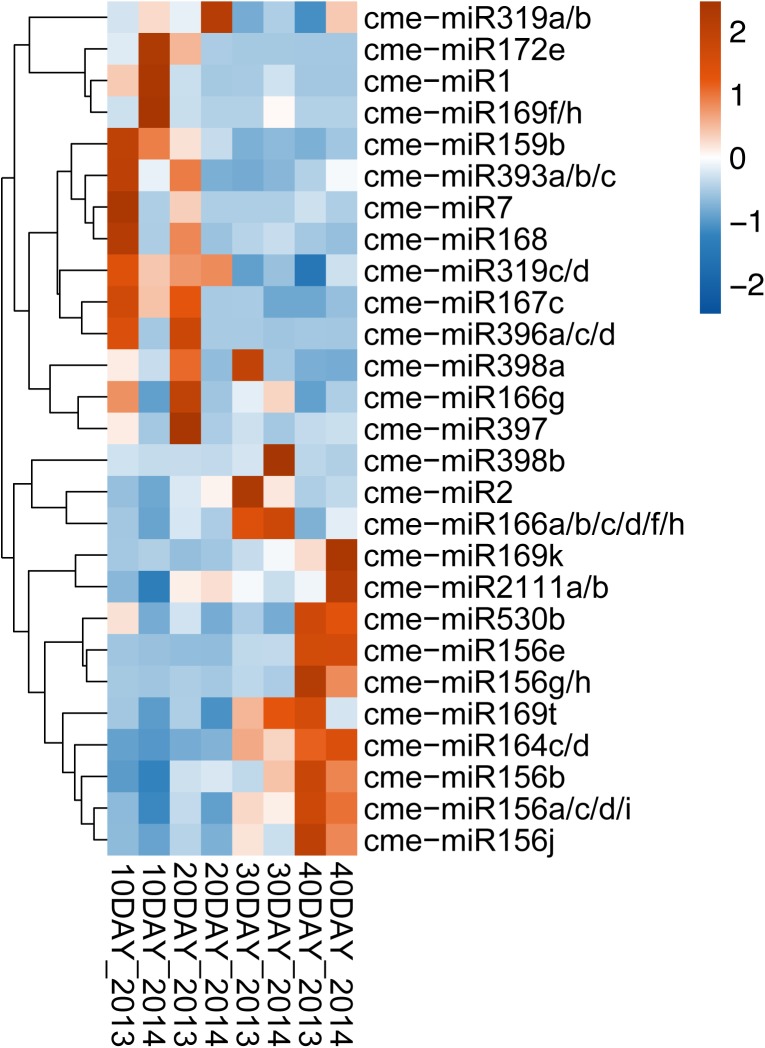
Differentially expressed Hami melon miRNAs during fruit development. The heatmap illustrates the expression profiles of differentially expressed miRNAs (log2TPM, y-axis) at the four developmental stages (x-axis). Colors range from blue to red, corresponding to low to high expressions. DAF represents days after flowering. 2013 represents the first batch samples collected in 2013. 2014 represents the second batch samples collected in 2014.

Differentially expressed miRNAs between the four developmental stages were determined using edgeR. There were 42 conserved miRNAs and 3 novel miRNAs pass the filter criteria (|fold change| >1.5 and *p* value <0.05) (S5 Table). According to the expression patterns, these developmentally regulated miRNAs were divided into three categories (Fig 3). The first category contained miRNAs that had relative high expression levels at early stages, then were down-regulated during fruit development. miR159, miR166g, miR167, miR168, miR169f/h, miR172, miR319, miR393, miR396, miR397, miR398a, miR1, and miR7 fell into this category. The second category comprised miRNAs that were up-regulated at 30DAF when compared with the early stages of fruit development, then down-regulated in 40DAF vs. 30DAF. miR166a/b/c/d/f/h, miR398b, and miR2 fitted into this category. The third category was composed of miRNAs that had low expression levels at early stages, then were up-regulated during fruit development. miR156, miR164, miR169k, miR169t, miR2111, miR530 belonged to this category.

**Fig 4:**
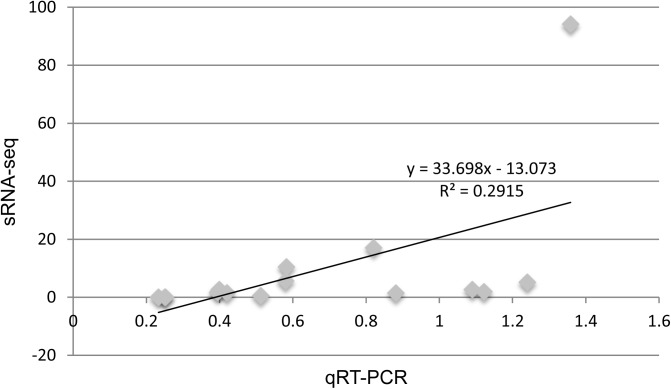
Validation of the expression profiles of conserved and novel miRNAs. The scatterplot of miRNA expression shows the correlation between small RNA sequencing (sRNA-seq) and qRT-PCR results.

To validate the transcriptome data, four conserved miRNAs (miR156a/c/d/i, miR156f, miR156g/h, and miR172e) and one novel miRNA (miR4) were selected for real-time quantitative reverse transcription (qRT)-PCR analysis. Due to lack of samples from the first batch, only samples from the second batch were used for qRT-PCR assay. For calculating the relative expression of each miRNA, the Ct value at 10DAF was used as a reference. Most tested miRNAs showed similar expression trends in sRNA-seq and qRT-PCR data, the correlation between these two methods was positive (*R* = 0.540, *P* <0.05) (Fig 4, S6 Table). This result illustrated that our high-throughput data was reliable.

### Prediction of miRNA targets

miRNAs commonly exert their functions by binding to the complementary target sites in mRNAs of their target genes. In this study, we used psRNATarget [42], a web-based program, for the prediction of putative miRNA targets. The transcriptome database of Hami melon fruit that contained 22,922 mRNA sequences [43] were used as a custom target database, and 113 conserved and nine novel mature miRNAs were used as a custom small RNA database. A total of 484 melon genes (606 transcripts) were predicted as putative targets of 29 conserved miRNA families (S7 Table). Of which, 67 genes (13.84%) were homologous to the previously reported targets of the same miRNA families in Arabidopsis (Table 3). Most of the conserved target genes have been validated in Arabidopsis, maize, rice, and poplar. The majority of these conserved targets (74.63%) encoded essential transcription factors, and the rest were homologous to plant proteins coded by Dicer-like protein, F-box proteins, ATP sulfurylases, laccases, copper/zinc superoxide dismutase, copper-zinc superoxide dismutase copper chaperone, inorganic phosphate transporter, and plantacyanin. The remaining 417 putative targets identified in this study were not conserved with other plant species. Among these targets, 195 genes (40.29%) exhibited no functional annotation. Similarly, 124 melon genes, including 168 transcripts, were predicted as the putative targets of nine Hami melon-specific miRNAs (S8 Table). Nearly half of these targets (49.19%) were not functionally annotated. The corresponding GO terms from predicted target genes were analyzed and the over-represented GO terms (*P* <0.05) were shown in S9 Table. Many target genes were related to “transcription, DNA-dependent”, “rRNA processing”, “oxidation reduction”, “signal transduction”, “regulation of transcription, DNA-dependent”, and “metabolic process” biological processes. We were not able to predict the targets for miR168, which maybe attributed to the insufficient mRNA sequences in Hami melon fruit transcriptome database.

**Table 3:** Conserved^a^ miRNA targets identified in Hami melon.

miRNA family	Target	Annotation
miR156	MELO3C025597	*SPL5*
	MELO3C017245	*SPL6*
	MELO3C002048	*SPL6*
	MELO3C002618	*SPL8*
	MELO3C009639	*SPL9*
	MELO3C005966	*SPL10*
	MELO3C002370	*SPL13A*
	MELO3C022318	*SPL13A*
	MELO3C014895	*SPL13A*
miR159	MELO3C018820	*MYB DOMAIN PROTEIN 101*
	MELO3C022282	*DUO POLLEN 1*, MYB protein
miR160	MELO3C025777	*ARF10*
	MELO3C019801	*ARF*16
	MELO3C011372	*ARF*17
miR162	MELO3C005929	*SUSPENSOR 1*, *DICER-LIKE 1*
miR164	MELO3C010555	*NAC DOMAIN CONTAINING PROTEIN 1*
	MELO3C025611	*CUP-SHAPED COTYLEDON 2*
	MELO3C001996	*NAC DOMAIN CONTAINING PROTEIN 100*
	MELO3C009855	*NAC DOMAIN CONTAINING PROTEIN 100*
	MELO3C017185	*NAC DOMAIN CONTAINING PROTEIN 100*
miR166	MELO3C007078	member of HD-ZIP III family, *PHAVOLUTA*
	MELO3C024446	member of HD-ZIP III family, *INCURVATA 4*
	MELO3C004887	member of HD-ZIP III family, *REVOLUTA*
	MELO3C025774	member of HD-ZIP III family, *REVOLUTA*
	MELO3C002807	member of HD-ZIP III family, *PHABULOSA 1D*
miR167	MELO3C007105	*ARF6*
	MELO3C002771	*ARF6*
	MELO3C025070	*ARF8*
miR169	MELO3C014590	CCAAT binding factor-HAP2-like protein, *NUCLEAR FACTOR Y SUBUNIT A1*
	MELO3C009551	CCAAT binding factor-HAP2-like protein, *NUCLEAR FACTOR Y SUBUNIT A10*
miR171	MELO3C021146	SCARECROW-like protein, *HAIRY MERISTEM 3*
	MELO3C013947	SCARECROW-like protein, *HAIRY MERISTEM 3*
	MELO3C017548	SCARECROW-like protein, *HAIRY MERISTEM 4*
	MELO3C017547	SCARECROW-like protein, *HAIRY MERISTEM 4*
miR172	MELO3C025726	*TARGET OF EARLY ACTIVATION TAGGED 1*
	MELO3C011722	*TARGET OF EARLY ACTIVATION TAGGED 1*
	MELO3C020848	*FLORAL MUTANT 2*, AP2
	MELO3C007572	*FLORAL MUTANT 2*, AP2
miR319	MELO3C019745	*TCP DOMAIN PROTEIN 10*
	MELO3C025629	*TCP4*
	MELO3C016092	*TCP4*
	MELO3C002754	*TCP2*
	MELO3C007121	*TCP2*
miR393	MELO3C015898	F-box protein, *TIR1*
	MELO3C014799	*AUXIN SIGNALING F-BOX 2*
miR394	MELO3C022422	a putative F-box protein, *LEAF CURLING RESPONSIVENESS*
miR395	MELO3C014280	*ATP SULFURYLASE 1*
	MELO3C006493	*ATP SULFURYLASE 1*
miR396	MELO3C010786	*GROWTH-REGULATING FACTOR 1*
	MELO3C009444	*GROWTH-REGULATING FACTOR 3*
	MELO3C004650	*GROWTH-REGULATING FACTOR 5*
	MELO3C024739	*GROWTH-REGULATING FACTOR 5*
	MELO3C025804	*GROWTH-REGULATING FACTOR 5*
	MELO3C015513	*GROWTH-REGULATING FACTOR 5*
	MELO3C006174	*GROWTH-REGULATING FACTOR 9*
miR397	MELO3C003228	*LACCASE-LIKE MULTICOPPER OXIDASE 4*
	MELO3C020569	*LACCASE-LIKE MULTICOPPER OXIDASE 4*
	MELO3C009189	*LACCASE-LIKE MULTICOPPER OXIDASE 4*
	MELO3C003213	*LACCASE 7*
	MELO3C014234	*LACCASE 11*
	MELO3C009250	*LACCASE 17*
	MELO3C009247	*LACCASE 17*
miR398	MELO3C015374	*COPPER/ZINC SUPEROXIDE DISMUTASE 1*
	MELO3C014007	*COPPER-ZINC SUPEROXIDE DISMUTASE COPPER CHAPERONE*
miR399	MELO3C012861	*PHOSPHATE TRANSPORTER 2*
miR408	MELO3C027302	*PLANTACYANIN*
	MELO3C008424	*PLANTACYANIN*

^a^ Conserved with *Arabidopsis thaliana*.

### Validation of miRNA-guided mRNA cleavage using 5’ rapid amplification of cDNA ends (RACE) [44]

**Fig 5:**
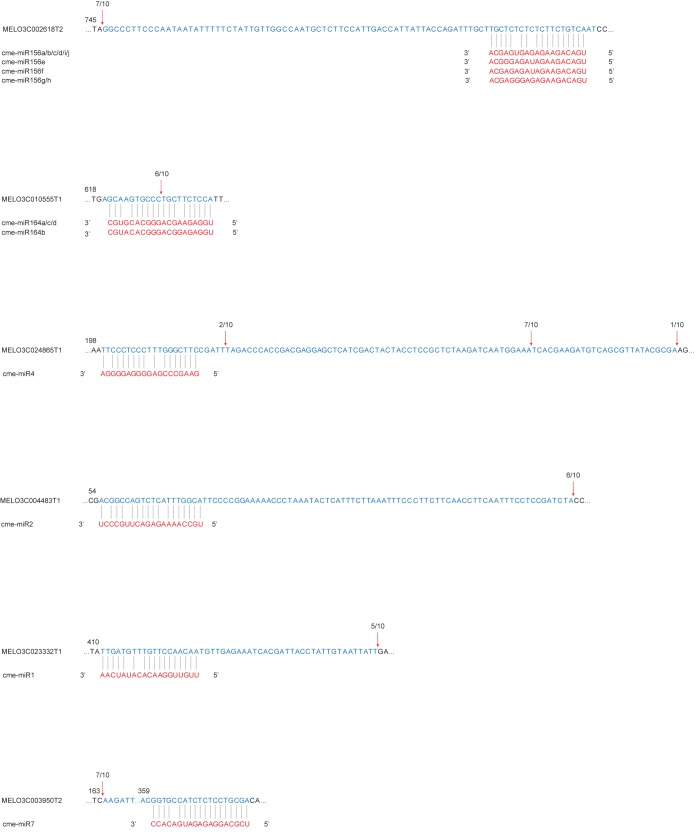
Verification of miRNA-guided mRNA cleavage using 5’ RACE. Partial sequences from target genes were aligned with the corresponding miRNAs. Each top strand (blue) represents a miRNA-homologous site in the target gene and each bottom strand (red) represents the aligned sequence of miRNA. Red arrows indicate the observed miRNA cleavage sites following 5’ RACE analysis, with the frequency of clones shown.

In this study, six melon transcripts of six predicted target genes were verified to be the targets of six Hami melon miRNAs. Sequencing of the 5’ RACE product of MELO3C010555T1 identified a precise cleavage at cme-miR164 binding site, between position 10 and 11 (Fig 5). MELO3C010555 encodes transcription factor NAC domain containing protein 1. MELO3C002618T2, MELO3C024865T1, MELO3C004483T1, MELO3C023332T1, and MELO3C003950T2 were validated to be targets of cme-miR156, cme-miR4, cme-miR2, cme-miR1, and cme-miR7, respectively, with a longer or shorter cleaved sequence. This result could be attributed to secondary siRNA in the 21-nucleotide register with the cleavage site for miRNAs as previously documented [45]. MELO3C002618 encodes transcription factor squamosa-promoter binding protein-like 8, MELO3C024865 encodes transcription factor NAC with transmembrane motif 1-like 6, MELO3C004483 encodes a homologous protein of TIP GROWTH DEFECTIVE 1, MELO3C023332 encodes a homologous protein of DEFECTIVE IN RNA-DIRECTED DNA METHYLATION 1, and MELO3C003950 encodes a homolog of COMPACT INFLORESCENCE 1.

## Discussion

### The expression profile of miRNAs in Hami melon fruit

In our previous study, the size, sugar content, and organic acid content of Hami melon fruit were closely related to the four developmental stages: 10DAF (young fruit), 20DAF (expanding), 30DAF (premature) and 40DAF (mature), and gene expression analysis revealed that the expression patterns were significantly altered from 20DAF to 30DAF [43]. Considering miRNAs exert important functions in the regulation of gene expression, this study is aiming to investigate the roles of miRNAs in Hami melon fruit development. A total of 54,553,424 sRNA raw reads were generated from the four developmental stages, 113 conserved miRNAs belonging to 30 miRNA families and nine novel miRNAs comprising nine miRNA families were identified. Consistently, the conserved miRNAs fast accumulated at the stages of 20DAF and 30DAF, implying their important roles in the phase transition of fruit ripening (S1 Table).

Many perennial fruit trees and shrubs have an irregular crop load from year to year, a high-yield crop is produced in one year (“on-year”), followed by a low-yield crop in the subsequent year (“off-year”). This phenomenon is called alternate or biennial bearing [46]. Some fruit crops are naturally biennial, but the regional conditions, such as climatic and edaphic managerial factors, can contribute to this problem [46, 47]. The expression of olive tree miRNAs has been reported to be regulated by alternate bearing, some conserved miRNAs showed significant differences in expression between on- and off-years [48]. Perennial fruit crops initiate flower buds for the following season’s crop in the current season, the alternation of high-yield and low-yield crops is caused by competition between the current season’s crop and the coming season’s flower buds. Hami melon is an annual plant, it completes its life cycle over the course of a single year. Therefore, there is no alternate bearing in Hami melon. However, the weather conditions and soil fertility do have an impact on melon yields and nutrient quality [49, 50]. miRNAs have diverse and important roles in plant growth and development, they are involved in signal transduction, protein degradation, response to environmental stress and pathogen invasion, and regulating their own biogenesis [51]. The environmental changes could affect the expression pattern of melon miRNAs. In the current study, among the determined conserved miRNAs, two miRNA families (miR159 and miR396) showed very high redundancies in each library (S1 Table). The expression of miR159a in the 10, 30, and 40DAF libraries constructed from the first batch samples was higher than the ones constructed from the second batch samples, while the expression of miR159a in the 20DAF library constructed from the first batch samples was much lower than the one constructed from the second batch samples. miR159b showed similar expression trends in the first batch and second batch datasets. miR396b and miR396e were expressed at higher levels in the 10 and 20DAF libraries constructed from the second batch samples than in the ones constructed from the first batch samples, but expressed at lower levels in the 30 and 40DAF libraries constructed from the second batch samples than in the ones constructed from the first batch samples. miR396a/c/d was expressed at lower levels in all libraries from the second batch than in the libraries from the first batch. Additionally, hierarchical cluster analysis revealed that Hami melon miRNAs in 20DAF libraries did not show a distinct expression pattern as those in 10DAF, 30DAF and 40DAF libraries did (Fig 2). The first and second batch melon plants were hand pollinated at May 2013 and 2014, respectively. The mature fruits of the first batch were harvested in mid June 2013, and the mature fruits of the second batch were harvested at the end of June 2014. The varied environmental conditions in the two cropping seasons (2013 and 2014) of this study could be an explanation for the differences in miRNA expression between the first and second batch datasets.

### Roles of miRNAs in Hami melon fruit development

The antagonistic activity of miR156 and miR172 in developmental transitions is one of the best understood and extensively reviewed miRNA network, which plays a highly conserved role in driving the progression through different developmental phases in both monocots and dicots [4, 52–57]. Karlova et al. [58] reported that *CNR*, a gene encoding one SPL protein, was a positive regulator of tomato fruit ripening, while a tomato *AP2* (*SlAP2a*) gene was shown to be a negative regulator of ripening. These two genes have been demonstrated to be actively modulated during fruit ripening, by miR156/157 and miR172, respectively [19]. More recently, the miR156-SPL module was found to control the initial steps of fleshy fruit development and determinacy [14]. In Hami melon, we also found a reverse expression pattern of cme-miR156 and cme-miR172 during fruit development, cme-miR156a-e/g-j were up-regulated and cme-miR172e was down-regulated at ripening stages when compared with the early stages of fruit development (Fig 3, S5 Table). Nine SPL proteins were predicted as targets of cme-miR156 and four AP2/trans-acting ethylene-responsive element binding protein (EREBP) transcription factors were predicted to be targets of cme-miR172 (Table 3, S7 Table), indicating miR156 and miR172 could play important roles in promoting developmental transition and fruit ripening. Magnesium chelatase catalyzes the first unique step toward chlorophyll formation in the tetrapyrrole biosynthesis pathway. Magnesium chelatase subunit I2 (CHLI2) contributes to the assembly of the Mg chelatase complex [59]. Pectate lyase enzyme activity obtained directly from banana pulp steadily increases during fruit ripening [60]. Antisense expression of a pectate lyase gene in strawberry resulted in significantly firmer fruits [61]. In this study, CHLI2 was predicted as the target of cme-miR156g/h, and a pectin lyase-like superfamily protein was predicted to be the target of cme-miR156j (S7 Table), suggesting that miR156 may regulate the fruit flesh color and softening of Hami melon through modulating CHLI2 and pectin lyase. Cationic amino acid transporter 8 (CAT8), belonging to amino acid polyamine choline transporter family, preferentially transports neutral and acidic amino acid analogs [62]. In Arabidopsis, CAT8 is developmentally regulated and appear to be involved in early seedling development [62]. In the current study, CAT8 was predicted to be the target of both cme-miR156a-e/g-i and cme-miR172e (S7 Table), implying that miR156 and miR172 may contribute to the early Hami melon fruit development by fine-tuning CAT8.

miR159 and miR319 are highly conserved and share extensive sequence similarity, however, they have largely non-overlapping effects in vivo [63, 64]. miR159 targets some *MYB* transcription factor genes involved in the regulation of vegetative growth, flowering time, anther development, seed shape and germination [65–67]. In contrast, miR319 mediates the onset of leaf senescence by regulating its main targets TCP transcription factor family [68]. Deep sequencing of tomato fruit development at 10 time-points showed that both miR159 and miR319 are most abundant during early fruit development [17]. Consistently, in Hami melon cme-miR159b and cme-miR319a/b/c/d both had high expression level at early development stages, and were down-regulated at the stages of fruit ripening (Fig 3, S5 Table). Two MYB proteins and five TCP transcription factors were predicted to be targets of cme-miR159 and cme-miR319, respectively (Table 3, S7 Table). miR159 and miR319 together with their conserved targets may play a role during the young fruit development. Glucosinolates are sulfur-rich, anionic natural products that are enzymatically hydrolyzed to produce several different products function as defense compounds and attractants with pungent or irritating taste and odor [69]. In Arabidopsis, brassinazole-resistant 1 (BZR1) and BRI1-EMS-suppressor 1 (BES1) are involved in the inhibition of glucosinolate biosynthesis by brassinosteroid [70]. Ascorbate-generated hydroxyl radicals can cause non-enzymatic solubilisation of polysaccharides in vitro, leading to the natural softening of fruit [71]. L-galactose-1-phosphate-phosphatase (VTC4) has been proposed to be a key regulator of ascorbic acid concentration during tomato fruit development and ripening [72]. In Hami melon, BES1/BZR1 homolog 4 and VTC4 were predicted as the targets of the down-regulated cme-miR159b (Fig 3, S5 and S7 Tables), indicating that miR159 maybe involved in the regulation of stress defense, fruit flavor and softening during fruit development.

miR164 regulates the formation of morphogenetic boundaries through directly targeting the NAC domain-encoding mRNAs [73, 74]. *CUP-SHAPED COTYLEDON* (*CUC)* genes function in establishing the boundary between meristematic, undifferentiated cells and those that are competent for differentiation [4]. Cell proliferation at the leaf margin determines leaf complexity, lack of CUC activity leads to simple leaves because of a precocious cessation of leaf margin growth [75]. ENHANCED VERY-LOW-FLUENCE RESPONSES 1 (EVE1) is required for the conversion of the early brassinosteroid precursor 24-methylenecholesterol to campesterol and plays a critical role in the general process of plant cell elongation [76, 77]. At present study, we predicted that cme-miR164 targeted five NAC transcription factors and EVE1 (Table 3, S7 Table). cme-miR164c/d were up-regulated during fruit development (Fig 3, S5 Table), indicating miR164 may negatively regulate cell proliferation and elongation at the early stages of fruit development through modulating its targets.

miR166 is known to have highly conserved targets that encode class III homeodomain-leucine zipper (HD-ZIP III) family members in a broad range of plant species [78]. Plant-specific HD-ZIP III transcription factors participated in many developmental processes [79, 80]. The high expression levels of miR166 at fruit ripening stages in both tomato [18] and banana [81] suggested this miRNA is a potentially important regulator of fruit ripening. At present study, five HD-ZIP III family members were predicted as the targets of cme-miR166 (Table 3, S7 Table). cme-miR166a/b/c/d/f/h were up-regulated in 30DAF vs. 10DAF, then down-regulated in 40DAF vs. 30DAF, while cme-miR166g was down-regulated in 40DAF vs. 30DAF (Fig 3, S5 Table). This finding confirms that miR166 has an important role in promoting fruit ripening and may contribute to the fine-tuning of HD-ZIP III proteins. Abscisic acid responsive elements-binding factor 2 (ABF2) is required for abscisic acid-mediated normal glucose response [82]. During Hami melon fruit development, the glucose content in fruit flesh is slightly increased at 30DAF comparing to 10 and 20DAF, then decreased at 40DAF [43]. ABF2 was predicted as the target of cme-miR166 a/b/c/d/f/g/h (S7 Table), suggesting miR166 maybe responsible for glucose accumulation and stress response during Hami melon fruit development by targeting ABF2.

Phosphorus is a critical macronutrient for plant growth and development. RING E3 ubiquitin ligase ATL80, a member of the Arabidopsis Tóxicos en Levadura (ATL) family, is shown to negatively affect the phosphorus mobilization and cold stress response in Arabidopsis [83]. In this study, we predicted cme-miR167c targeted *ATL80* gene (S7 Table). cme-miR167c was down-regulated at the stages of fruit ripening (Fig 3, S5 Table), indicating that miR167 may play an essential role in phosphorus transport and cold stress tolerance at the early stages of Hami melon fruit development, by regulating ATL80.

miR169 targets members of the *NUCLEAR FACTOR Y*, *subunit A* (*NF-YA*) gene family to regulate plant development and stress responses [84–86]. The expression level of miR169 is very low at the fruit ripening stages of tomato [18] and banana [81]. At present study, cme-miR169f/h had a low level at all fruit development stages and was down-regulated in 20DAF vs. 10DAF and 40DAF vs. 10DAF, while cme-miR169k/t were up-regulated in 40DAF vs. 10DAF and 40DAF vs. 20DAF (Fig 3, S5 Table). Two *NF-YA* genes were predicted as the targets of cme-miR169 (Table 3, S7 Table), suggesting miR169-NF-YA modules may have multiple roles in response to stress signaling during Hami melon fruit development.

It is known that the ancient miRNA miR396 controls cell proliferation in Arabidopsis leaves through its conserved target GROWTH-REGULATING FACTORs (GRFs) [87]. Recent study showed that miR396 modulates fruit enlargement and weight via GRF transcription factors in tomato [15]. In this study, seven GRF transcription factors were predicted to be targets of cme-miR396 (Table 3, S7 Table). cme-miR396a/c/d were down-regulated at ripening stages comparing to the early stages of fruit development (Fig 3, S5 Table), which suggested that miR396-GRF modules maybe required for young fruit development and enlargement in Hami melon.

miR393 regulation of F-box proteins is important for auxin-dependent plant development and for response to environment [88–91]. Down-regulation of TRANSPORT INHIBITOR RESPONSE 1 (TIR1) plays a critical role at the flower-to-fruit transition in tomato [92]. Using high-throughput degradome library sequencing, miR393 is shown to regulate the expression of TIR1 and its homologues during tomato fruit set [19]. In litchi, miR393 was reported to have an important role in fruit senescence through regulating its target auxin signaling F-box 2 (AFB2) [93]. In Hami melon, we predicted that cme-miR393 targeted both *TIR1* and *AFB2* genes (Table 3, S7 Table). cme-miR393a/b/c was down-regulated in 30DAF vs. 10DAF (Fig 3, S5 Table), implying that miR393 may be involved in Hami melon fruit set and negatively regulate fruit ripening and senescence by modulating the expression of F-box proteins.

miR397 and its target laccases have been reported to co-regulate the lignin content in poplar [94] and Arabidopsis [95]. Lignin is one of the organic polymers that strengthen plant cell walls. In strawberry, the lignin content is closely correlated with fruit firmness [96, 97]. Beta-galactosidase is one of the key enzymes in fruit ripening. The beta-galactosidase isozymes in muskmelon has been reported to be able to hydrolyze the pectin and hemicellulose in vitro from its fruit cell walls [98]. At present study, seven laccases and beta-galactosidase 7 were predicted as the targets of cme-miR397 (Table 3, S7 Table). cme-miR397was down-regulated in 30DAF vs. 20DAF (Fig 3, S5 Table), indicating that miR397 may regulate fruit firmness and softening during Hami melon fruit development.

miR398 mediates plant responses to abiotic and biotic stresses through regulating the expression of its target genes, *Cu or Zn superoxide dismutase* (*CSD*) [99–101] and *the copper chaperone for superoxide dismutase* (*CCSD*) [102, 103]. Over-expression of Arabidopsis blue copper binding protein (BCB) in transgenic lines caused an accumulation of lignin and a decrease of aluminium-stress-induced lipid peroxides [104]. Associated with tolerant to chilling and freezing 1, BCB was reported to promote lignin biosynthesis in response to cold stress [105]. In this study, one *CSD* gene and one *CCSD* gene were predicted as the targets of cme-miR398b and two *BCB* genes were predicted to be the targets of cme-miR398a/b (Table 3, S7 Table). cme-miR398a was down-regulated at 40DAF comparing with the other three stages, while cme-miR398b was up-regulated in 30DAF vs. 20DAF, then down-regulated in 40DAF vs. 30DAF (Fig 3, S5 Table). This finding indicated that miR398 participate in Hami melon fruit development in two different manners, both miR398a and b may modulate the lignin formation in response to various environmental stresses during fruit development, and miR398b maybe linked to direct responses to stresses and the downstream signaling processes at the ripening stages.

miR2111 was induced by phosphate deficiency in Arabidopsis [106]. The kelch-domain containing proteins with unknown function were predicted as the targets of phosphate-responsive miR2111 [106, 107]. At present study, cme-miR2111a/b were up-regulated during Hami melon fruit development (Fig 3, S5 Table), and we predicted that cme-miR2111a/b targeted a homologous of lysophosphatidyl acyltransferase 5 (S7 Table). Phospholipid/glycerol acyltransferase is involved in the synthesis of phospholipids, the major lipid component of the most cellular membranes. This finding suggested that miR2111 might mediate the membrane biogenesis during fruit growth and ripening by regulating its target phospholipid/glycerol acyltransferase.

An inactive homolog of class III chitinases was reported to accumulate in immature banana fruits and possibly serve as a source of amino acids for the synthesis of ripening-associated proteins [108]. In this study, chitinase A, a class III chitinase, was predicted as the target of cme-miR530b (S7 Table). cme-miR530b was up-regulated at 40DAF comparing with the other three stages (Fig 3, S5 Table), implying that miR530 is probably involved in the ripening process of Hami melon fruit by regulating the expression of chitinase A.

Senescence-associated E3 ubiquitin ligase 1 (SAUL1) prevent premature senescence in Arabidopsis [109]. Down-regulation of SAUL1 enhanced abscisic acid (ABA) biosynthesis [109]. ABA plays an important role in fruit softening and ripening in strawberry [110, 111] and tomato [112]. DEFECTIVE IN RNA-DIRECTED DNA METHYLATION 1 (DRD1) is a putative chromatin remodeling protein, belonging to a plant-specific subfamily of SWITCH 2/SUCROSE NONFERMENTING 2-like proteins [113, 114]. DRD1 is positively associated with leaf senescence in Arabidopsis [115]. ERECTA-LIKE (ERL) 1, together with ERECTA and ERL2 promotes intercellular communication that is essential for coordinated cell proliferation and organ growth [116]. In Hami melon, SAUL1, DRD1, and ERL1 were predicted to be the targets of cme-miR1 (S8 Table). cme-miR1 was down-regulated during fruit development (Fig 3, S5 Table), implying that cme-miR1 may participate in ABA signaling and fruit growth, ripening, and senescence by modulating its targets.

TIP GROWTH DEFECTIVE 1 (TIP1) plays a role in both tip and diffuse cellular growth, and affects organ size throughout the plant [117–119]. In this study, we predicted that cme-miR2 targeted a homologous protein of TIP1 (S8 Table). cme-miR2 was up-regulated in 30DAF vs. 10DAF and down-regulated in 40DAF vs. 30DAF (Fig 3, S5 Table), indicating that cme-miR2 may modulate the cell growth and fruit size of Hami melon through negatively regulating TIP1.

Compact inflorescence (CIF) 1 plays a vital role in regulating the phase transition from juvenile to adult, the growth pattern of adult vegetative tissues is dramatically affected in *cif1* mutant plants when combined with the dominant CIF2 modifier allele [120, 121]. In the current study, we predicted that cme-miR7 targeted CIF1 (S8 Table). cme-miR7 was down-regulated in 30DAF vs. 10DAF (Fig 3, S5 Table), suggesting cme-miR7 maybe involved in the phase transition from young fruit to mature fruit during Hami melon fruit development through targeting CIF1.

### A miRNA-transcription factor network may contribute to the regulation of Hami melon fruit ripening

Fruit ripening is mediated by multiple transcriptional cascades, in which the upstream signaling and transcription regulators are induced to activate or repress the downstream ripening pathways including ethylene, carotenoids and cell wall metabolism. NAC, MYB, AP2 domain, SPL, HD-ZIP homeobox protein are the common classes of transcription factors that are differentially expressed during tomato [122] and melon [123] fruit ripening. In tomato, a NAC gene, *NON-RIPENING*, positively regulates fruit ripening [124]. Another NAC transcription factor, SINAC4, also functions as a positive regulator of fruit ripening by affecting ethylene synthesis and carotenoid accumulation [125]. A SPL protein, CNR, is critical for normal fruit ripening [126]. The tomato AP2a transcription factor negatively regulates ethylene biosynthesis and carotenoid accumulation [58, 127], while CNR functions upstream of AP2a and positively regulate its expression [58]. A HD-Zip homeobox protein, LeHB-1, plays an important role in the control of fruit ripening [128]. In Hami melon, we predicted that cme-miR156 targeted nine SPL proteins, cme-miR172 targeted four AP2/EREBP transcription factors, cme-miR164 targeted five NAC transcription factors, and cme-miR166 targeted five HD-ZIP III family members. cme-miR4 was detected in sRNA libraries in this study, but excluded in the differentially expressed miRNAs because of its distinct expression trends between the first and second batches (S3 Table). One NAC domain protein was predicted as the target of cme-miR4 and validated through our 5’ RACE experiment (Fig 5). MiR156-SPL, miR164-NAC, cme-miR4-NAC, miR166-HD-ZIP III may positively regulate fruit ripening, while miR172-AP2 could negatively regulate the ripening process. The miR156-SPL and miR172-AP2 modules are possibly part of a negative feedback loop in the regulation of Hami fruit ripening.

## Conclusions

In the present study, we performed a close examination of the dynamic regulation of miRNAs during sequential stages of Hami melon fruit development. Our results revealed that specific miRNAs were differentially regulated during fruit development, and therefore may play important roles in fruit growth and ripening. All the related regulatory events maybe contributing in a balanced manner to the process of normal Hami fruit development and ripening. The findings of this study could be used for further research and applications in breeding practices. Ripening-associated transcription factors, NAC, SPL, AP2, HD-ZIP III, and ARF, were predicted as the targets of developmentally regulated miRNAs in Hami melon, further investigation on how miRNAs and ripening-associated transcription factors interact during the programmed process of fruit ripening may help us find the key factors that determine the long shelf life of Hami melon.

## Materials and methods

### Plant materials

‘Flavor No. 4’, a medium-maturing variety of Hami melon with unique sweet and sour taste, was cultivated in the greenhouse at Institute of Vegetables and Flowers, Chinese Academy of Agricultural Sciences (Beijing, China). When female flowers (one per plant) open, they were hand pollinated in the same day to precisely determine the stage of development. Fruits at four typical stages of development: 10DAF (young fruit), 20DAF (expanding), 30DAF (premature) and 40DAF (mature) were harvested. Sucrose accumulation can be used as a marker for the onset of fruit ripening in melon [123]. In Hami melon, the sucrose content dramatically increased at 30DAF [43], suggesting 30DAF and 40DAF represent the fruit ripening stages. The fruit samples were harvested in two batches, the first batch samples (three fruits from three different plants at each developmental stage) were collected in 2013 and the second batch samples (one fruit from one plant at each developmental stage) were collected in 2014. The first batch melon plants were hand pollinated at May 2013 and the mature fruits were harvested in mid June 2013. While the second batch plants were hand pollinated at May 2014 and the mature fruits were harvested at the end of June 2014. Flesh mesocarp was taken from the center-equatorial portion of each fruit, and then chopped into small pieces. Sliced flesh mesocarp samples were snap-frozen in liquid nitrogen and kept at -80°C until required.

### RNA extraction and small RNA sequencing

Total RNA was extracted by using TRIzol reagent (Invitrogen, Carlsbad, CA, USA) according to the manufacturer’s instructions, and RQ1 DNase (Promega, Madison, WI, USA) was used to remove contaminating genomic DNA. The quality and quantity of the purified RNA was monitored at the ratios of A260/A280 and A260/230 on SmartSpec Plus Spectrophotometer (BioRad, Philadelphia, PA, USA). RNA integrity was further verified by 1.5% agarose gel electrophoresis.

For sRNA sequencing, total RNA was extracted from flesh mesocarp samples collected from each melon fruit. For the first batch samples, the RNA extractions from the same developmental stage were mixed together. Three μg of total RNA from each developmental stage was used for sRNA cDNA library preparation with Balancer NGS Library Preparation Kit (Gnomegen, San Diego, CA, USA) based on manufacturer’s instruction. Whole library was applied to 10% native PAGE gel electrophoresis and bands corresponding to miRNA insertion were cut and eluted. After ethanol precipitation and washing, the purified small RNA libraries were quantified with QubitFluorometer (Invitrogen, Carlsbad, CA, USA). The four small RNA libraries from the first batch were used for cluster generation and applied to Illumina GAIIx (Illumina, San Diego, CA, USA) 73 nt single-end sequencing. The four small RNA libraries from the second batch were applied to Illumina NextSeq 500 (Illumina, San Diego, CA, USA) 76 nt pair-end sequencing. All eight sRNA libraries in two separate batches were sequenced to obtain independently repeated data.

### Conserved and novel miRNA identification

After the completion of sequencing, raw reads were processed by FASTX-Toolkit (Version 0.0.13) to obtain reliable clean reads. During this procedure, adaptor sequences, low quality tags and sequences shorter than 18 nt were removed. Based on the length of the mature miRNA and adapter length, RNAs smaller than 18 nt and greater than 30 nt in length were excluded from the further analysis. The obtained high-quality clean reads were subsequently searched against the Rfam database (version 12.0) using Bowtie [129]. The matches to rRNAs and tRNAs were excluded. Next, the remaining unique sequences were aligned against miRBase (v21, July 2014) [33] using Bowtie (one mismatch allowed). The matched sRNA sequences were considered to be conserved miRNAs. Unaligned sequences were potential candidates for novel miRNAs. To identify novel miRNAs, the unique sequences were aligned to MELONOMICS reference genome sequence (v3.5) [38] using algorithm miRDeep [39]. Mfold (http://unafold.rna.albany.edu) [40] was employed to explore the secondary structures of the putative precursors utilizing default parameters. Precursors that met structural miRNA criteria were kept for further analysis.

### Expression analysis of the identified miRNAs

To investigate the expression profiles of identified miRNAs in the eight sRNA libraries, the frequency of miRNA counts were normalized to TPM using the following formula: normalized expression = actual read count / total read count × 10^6^. Each batch was considered as a biological replicate set. Differentially expressed miRNAs between the four developmental stages were analyzed using the software edgeR [130]. A combination of |fold change| >1.5 and *p*-value <0.05 were used as the threshold to determine the significance of differentially expressed miRNAs.

### Real-time quantitative reverse transcription (qRT) -PCR

Because fruit samples from the first batch were used for both RNA sequencing and small RNA sequencing, and the RNA sequencing data have been published last year [43]. The remaining volume of RNA samples from the first batch was not sufficient for qRT-PCR experiments, only samples from the second batch were used for qRT-PCR assay. One microgram of total RNA was reverse transcribed using M-MLV Reverse Transcriptase according to the manufacturer’s protocol (Promega, Madison, WI, USA). We selected U6 snRNA as the internal control. The stem-loop qRT-PCR primers for mature miRNAs were designed by us (S10 Table), the stem-loop qRT-PCR primers for U6 were designed and provided by RIBOBIO (Guangzhou RIBOBIO Co., Ltd, Guangzhou, China). Real-time monitoring of PCR was performed using SYBR Green Realtime PCR Master Mix (Toyobo, Osaka, Japan) and LightCycler 480 (Roche, Indianapolis, IN, USA). The reaction was 20 μl system containing 1 μl of diluted cDNA (equivalent to 100 pg of total RNA), 10 μl of 2 × SYBR green reaction mix, and 5 pmol of the forward and the reverse primers. The qRT-PCR was conducted in triplicate for 1 min at 95°C, followed by 40 cycles of 15 s at 95°C, 20 s at 60°C and 20 s at 72°C. The 2^-ΔΔCt^ method was utilized to calculate the fold change in miRNA expression [131]. For calculating the relative expression of each miRNA, the Ct value at 10DAF was used as a reference.

### miRNA target prediction and validation

The putative targets of identified miRNAs were predicted using psRNATarget with default settings (http://plantgrn.noble.org/psRNATarget/) [42]. The custom transcript databases include 22,922 mRNA sequences identified from the transcriptome database of Hami melon fruit [43]. All predicted target genes were evaluated by scoring system, and sequences were considered to be putative miRNA targets if a penalizing score was no more than 3 points. The putative targets were annotated using BLASTX against the non-redundant (Nr) and Arabidopsis Information Resource (TAIR) database.

Two μg total RNA from equally mixed four RNA extractions of 10DAF, 20DAF, 30DAF and 40DAF was used to synthesize 5’-RACE-ready cDNAs with the 5’-Full RACE Kit (Takara Bio Inc., Otsu, Shiga, Japan) according to the manufacturer’s instructions. The final PCR product was extracted and purified from a 2% agarose gel, cloned into pEASY-T1 Vector (Beijing TransGen Biotech Co., Ltd, Beijing, China), and plasmid DNA from 10 different colonies was sequenced. The outer and inner gene specific primers were listed in S10 Table.

### Accession numbers

The accession number for the small RNA sequence data reported in this paper is GEO: GSE77127.

## Supporting information

S1 Fig Comparison of our sRNA reads data with the previously published melon sRNA data [20]. 1–5, 1 ≤ read count < 5; 5–10, 5 ≤ read count < 10; 10–15, 10 ≤ read count < 15; 15–20, 15 ≤ read count < 20; 20–25, 20 ≤ read count < 25; 25–30, 25 ≤ read count < 30; > = 30, 30 ≤ read count. DAF represents days after flowering. 2013 represents the first batch samples collected in 2013. 2014 represents the second batch samples collected in 2014.
(PDF)

S2 Fig Length distribution pattern of annotated and unannotated small RNA sequences in different libraries. nt, nucleotides. DAF represents days after flowering. 2013 represents the first batch samples collected in 2013. 2014 represents the second batch samples collected in 2014.
(PDF)

S3 Fig Length distribution of the miRNAs identified by aligning to known miRNAs in miRBase or computational methods. nt, nucleotides. DAF represents days after flowering. 2013 represents the first batch samples collected in 2013. 2014 represents the second batch samples collected in 2014.
(PDF)

S4 Fig The secondary structures of Hami melon-specific miRNAs predicted by Mfold. (PDF)

S1 Table Identification of conserved miRNAs in Hami melon. (XLS)

S2 Table The precursor sequence of conserved miRNAs. (XLS)

S3 Table Identification of Hami melon-specific miRNAs. (XLS)

S4 Table The precursor sequence of Hami melon-specific miRNAs. (XLS)

S5 Table Differentially expressed miRNAs during Hami melon fruit development. m. value = log_2_(fold change). “the absolute value of m. value >log_2_1.5 and p. value <0.05” was set as the threshold to determine the significance of differentially expressed miRNAs.
(XLS)

S6 Table Expression profiles of miRNAs measured by small RNA sequencing and stem-loop qRT-PCR. Relative expression level was calculated using TPM/TPM-10DAF. qRT-PCR data were averaged using the results from three independent experiments.
(XLSX)

S7 Table The targets of Hami melon conserved miRNAs. (XLS)

S8 Table The targets of Hami melon-specific miRNAs. (XLSX)

S9 Table GO enrichment analysis of the target genes. Sheet 1: Cellular component category. Sheet 2: Molecular function category. Sheet 3: Biological process category.
(XLS)

S10 Table The primers used in this study. (XLSX)
