# Supplementary figures and images for: Genome-wide identification of Hami melon miRNAs with putative roles during fruit development
Source: PLoS One. 2017 Jul 24;12(7):e0180600. doi: 10.1371/journal.pone.0180600 (PMC5524408; doi:10.1371/journal.pone.0180600)

S1 Fig

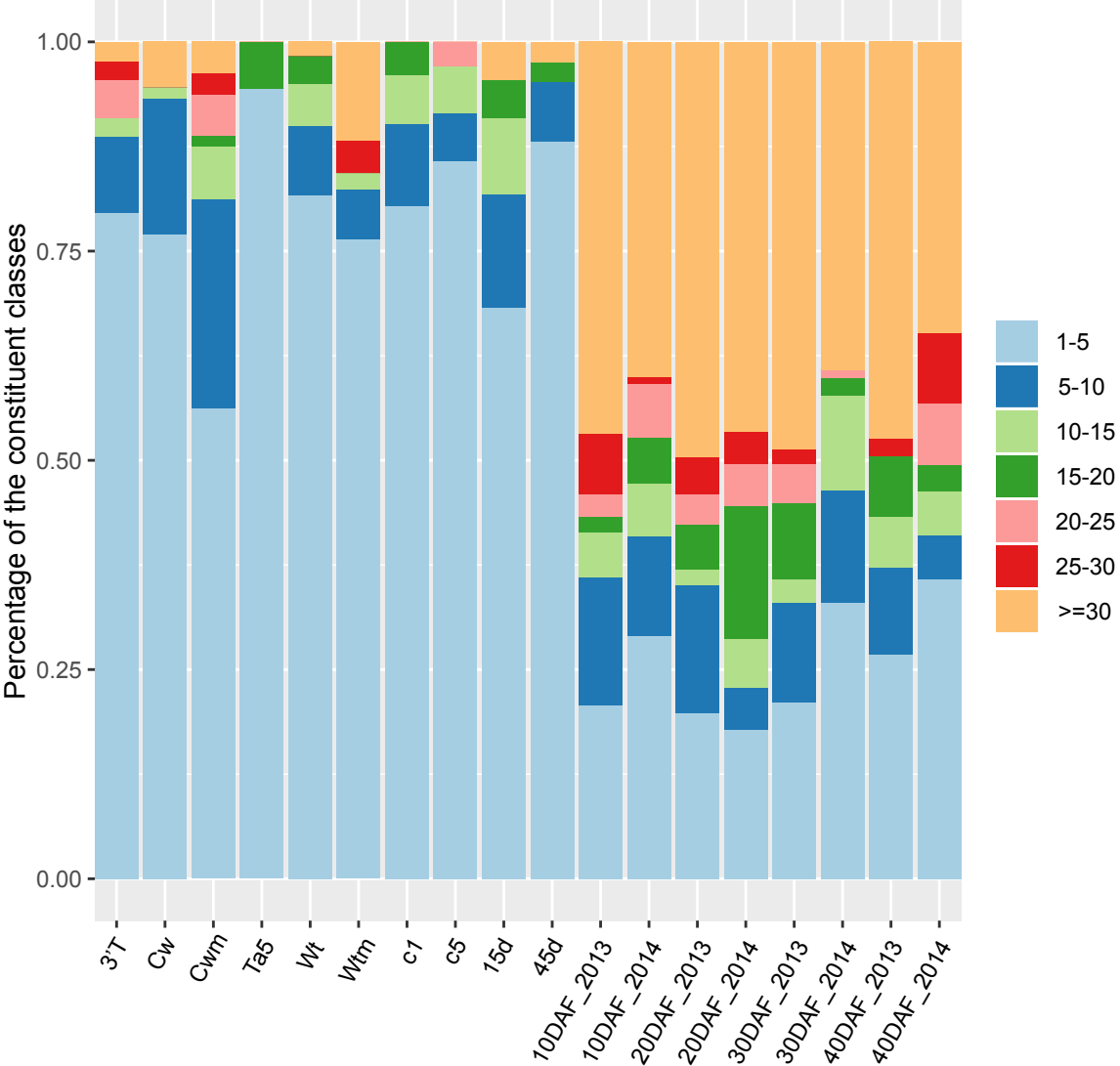

Supplement: S1 Fig — 1–5, 1 ≤ read count < 5; 5–10, 5 ≤ read count < 10; 10–15, 10 ≤ read count < 15; 15–20, 15 ≤ read count < 20; 20–25, 20 ≤ read count < 25; 25–30, 25 ≤ read count < 30; > = 30, 30 ≤ read count. DAF represents days after flowering. 2013 represents the first batch samples collected in 2013. 2014 represents the second batch samples collected in 2014. (PDF) [file pone.0180600.s001.pdf]

S2 Fig

Others

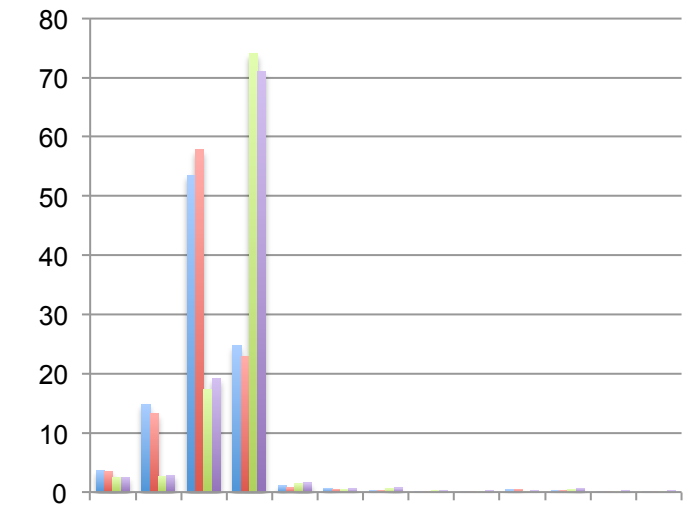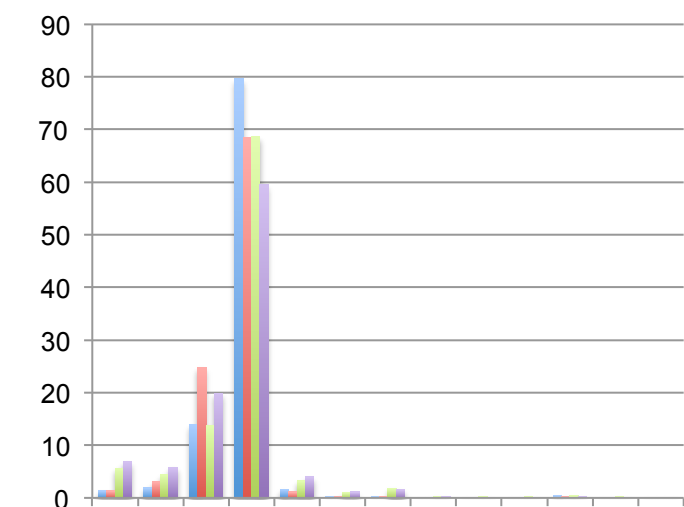

miRNA

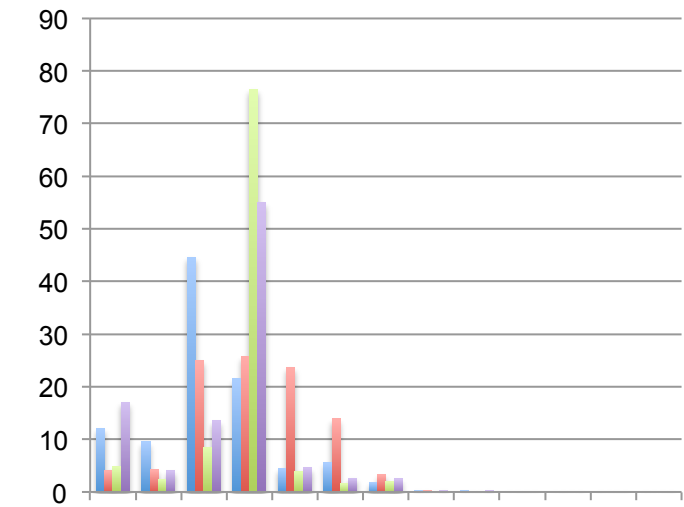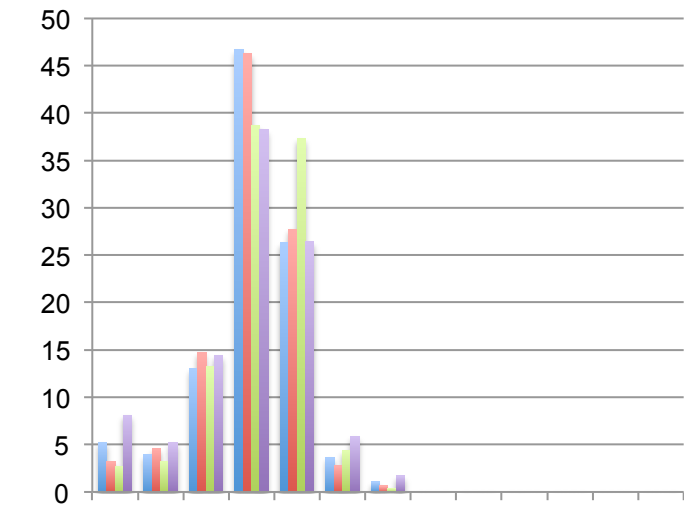

rRNA

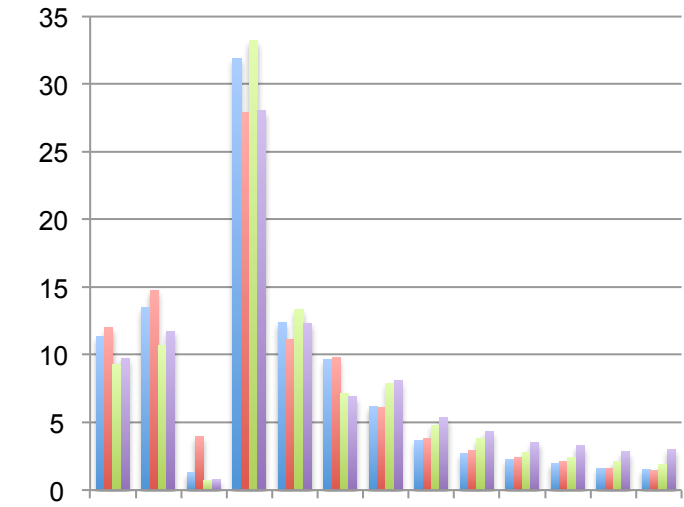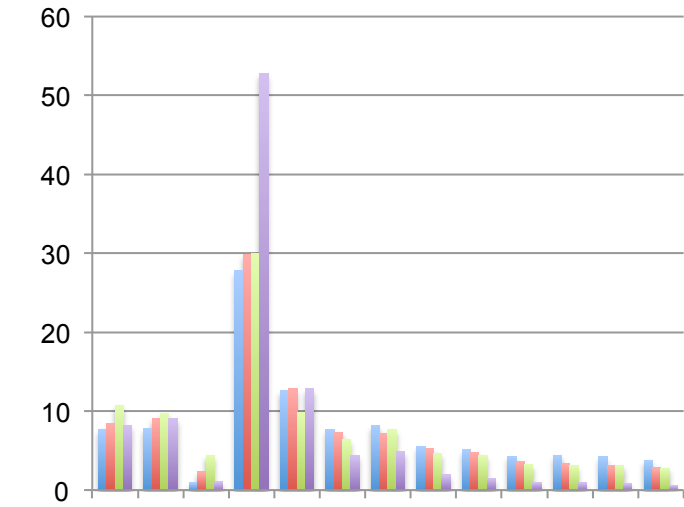

tRNA

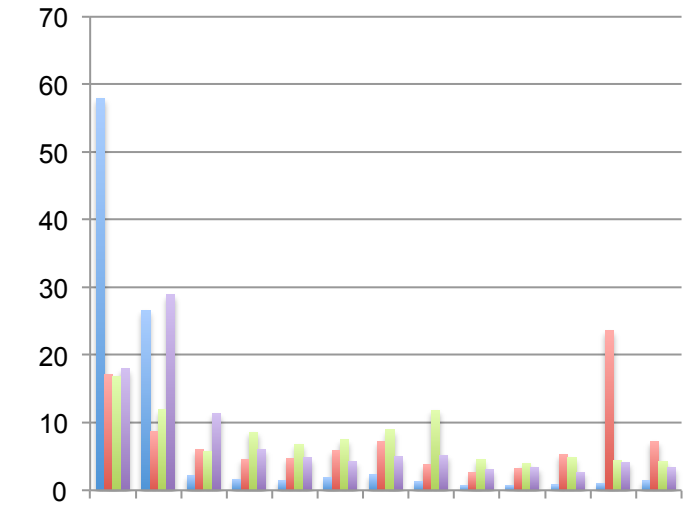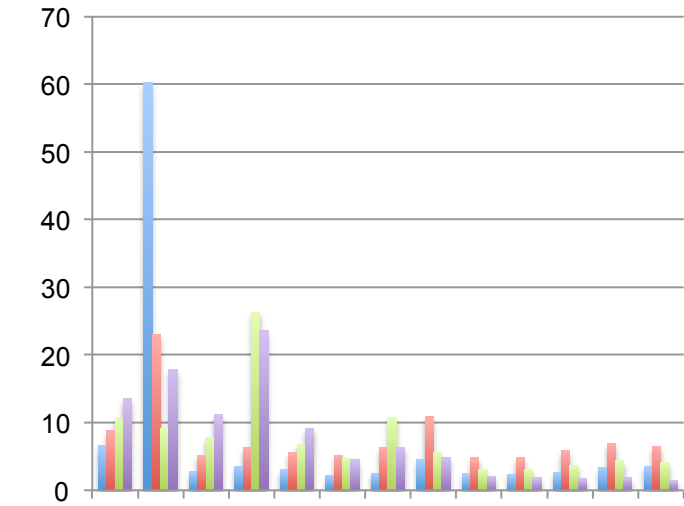

Unannotated

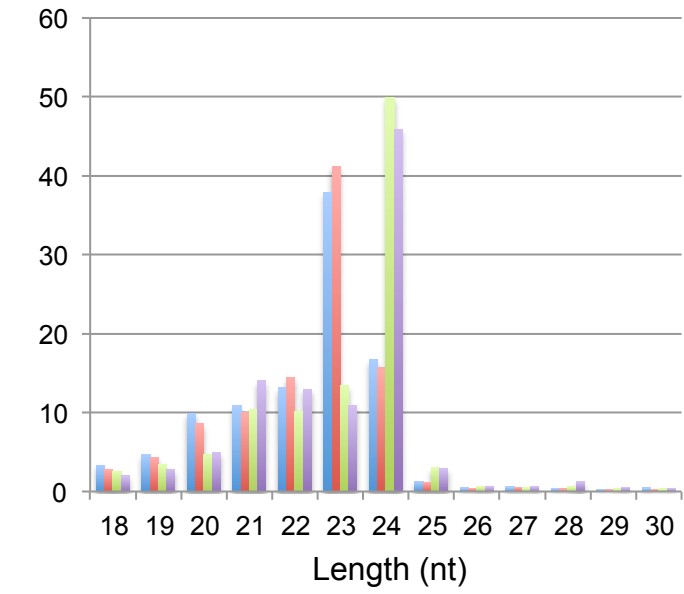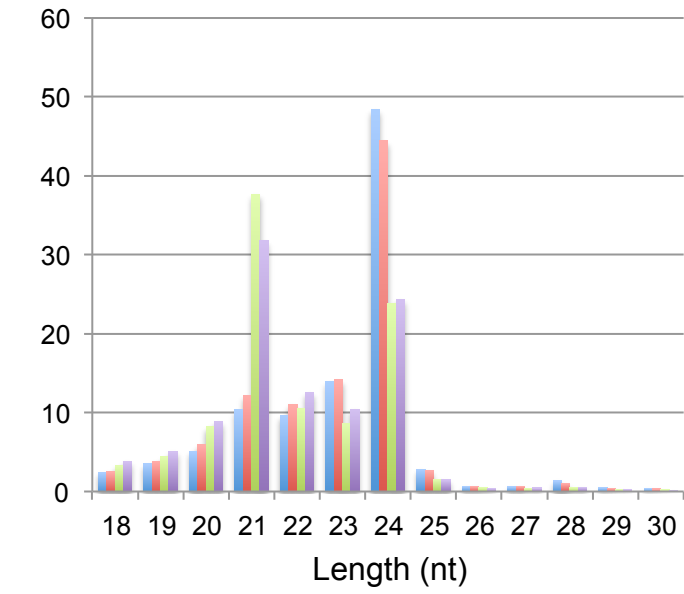

10DAF\_2013 20DAF\_2013 30DAF\_2013 40DAF\_2013

10DAF\_2014 20DAF\_2014 30DAF\_2014 40DAF\_2014

Supplement: S2 Fig — DAF represents days after flowering. 2013 represents the first batch samples collected in 2013. 2014 represents the second batch samples collected in 2014. (PDF) [file pone.0180600.s002.pdf]

S3 Fig

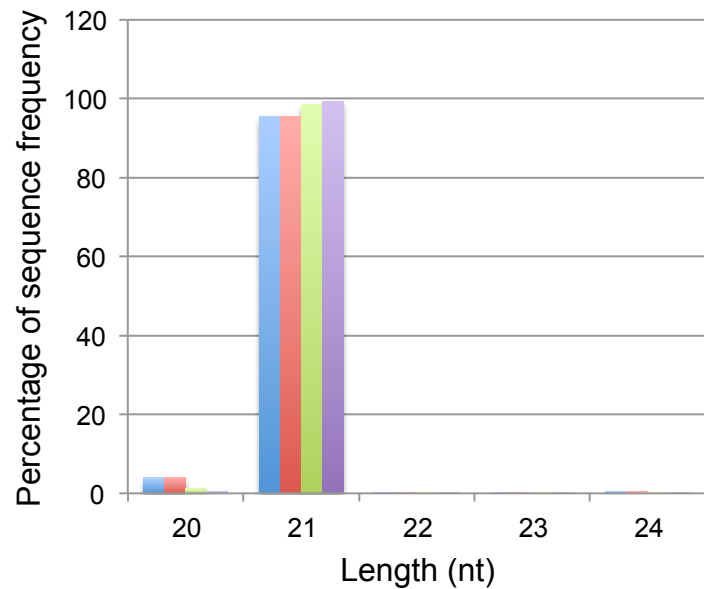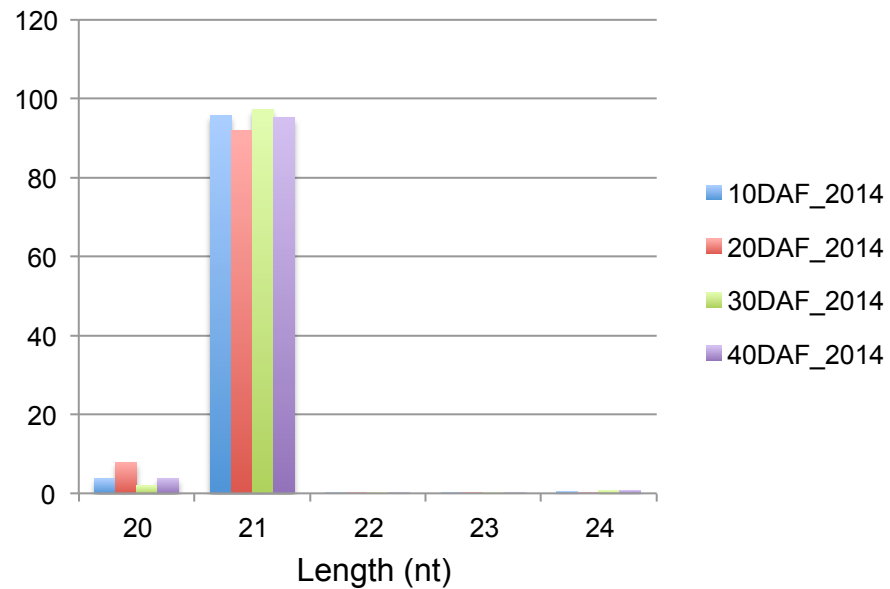

S3 Fig

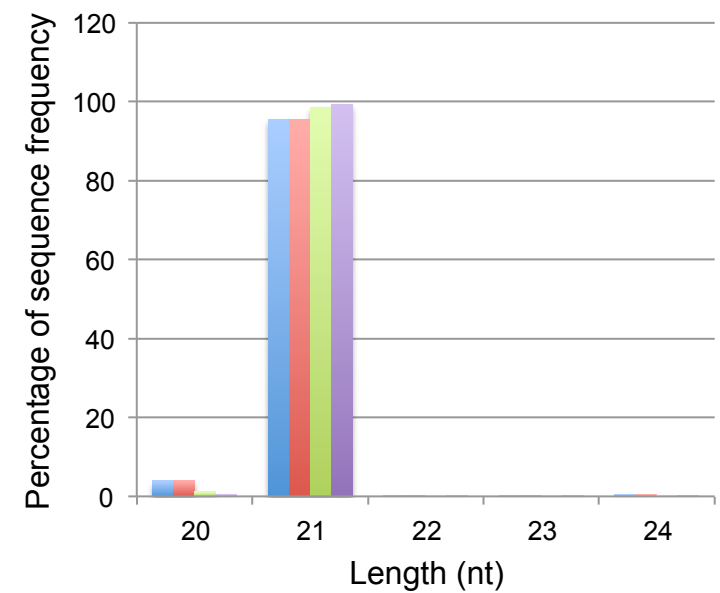

10DAF\_2013  
20DAF\_2013  
30DAF\_2013  
40DAF\_2013

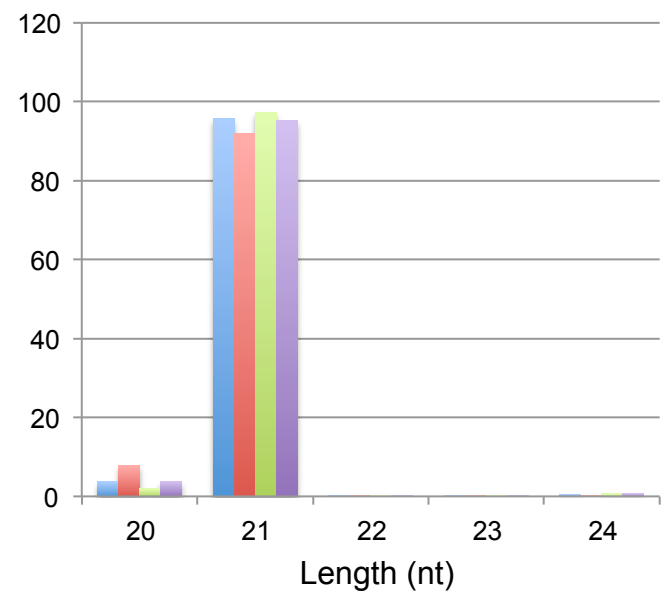

10DAF\_2014  
20DAF\_2014  
30DAF\_2014  
40DAF\_2014

Supplement: S3 Fig — DAF represents days after flowering. 2013 represents the first batch samples collected in 2013. 2014 represents the second batch samples collected in 2014. (PDF) [file pone.0180600.s003.pdf]
