# Supplementary material for: Genome-wide identification of Hami melon miRNAs with putative roles during fruit development
Source: PLoS One. 2017 Jul 24;12(7):e0180600. doi: 10.1371/journal.pone.0180600 (PMC5524408; doi:10.1371/journal.pone.0180600)
Supplement: S4 Fig — (PDF) [file pone.0180600.s004.pdf]

# S4 Fig

Output of sir_graph (©)
mfold_util 4.7

Created Tue Nov 22 03:00:50 2016

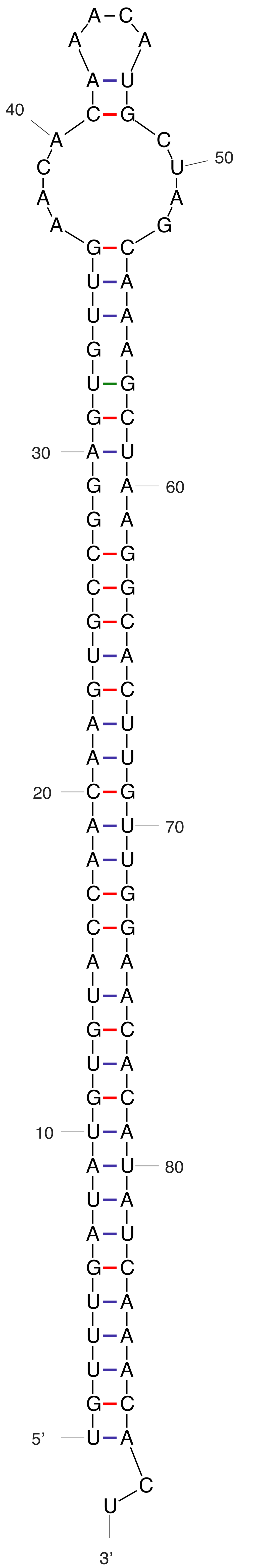


*dG = -47.70 [Initially -47.70] cme-miR1*

Output of sir_graph (©)
mfold_util 4.7

Created Tue Nov 22 02:42:20 2016

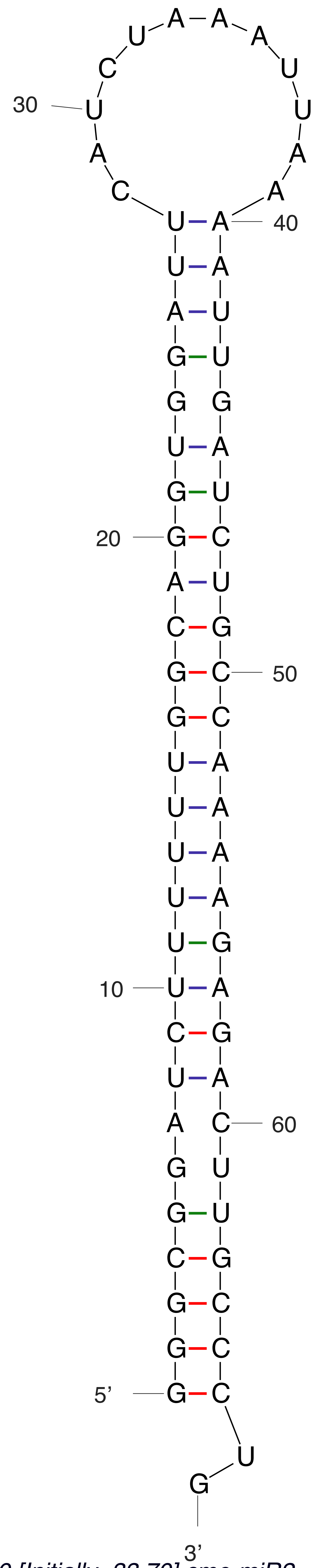


*dG = -33.70 [Initially -33.70] cme-miR2*

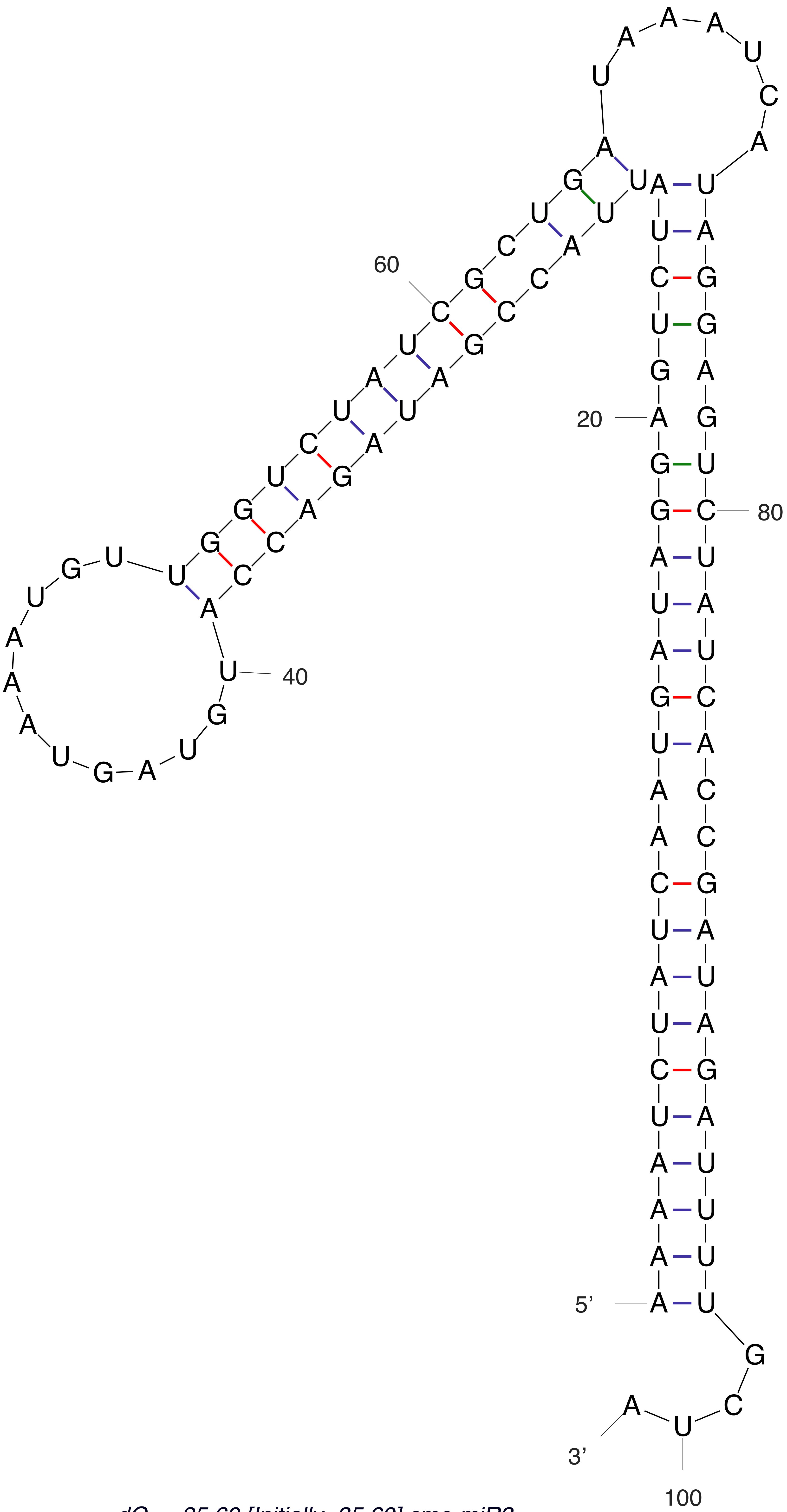


*dG = -35.60 [Initially -35.60] cme-miR3*

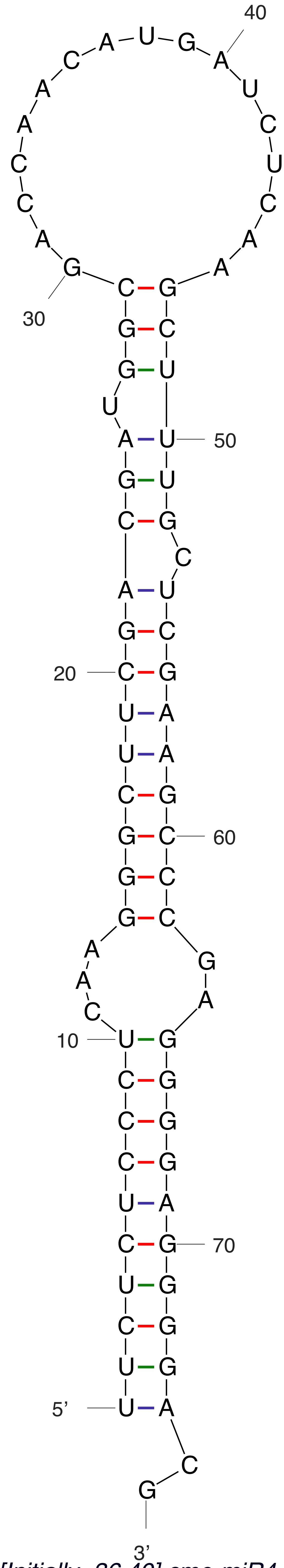


*dG = -36.40 [Initially -36.40] cme-miR4*

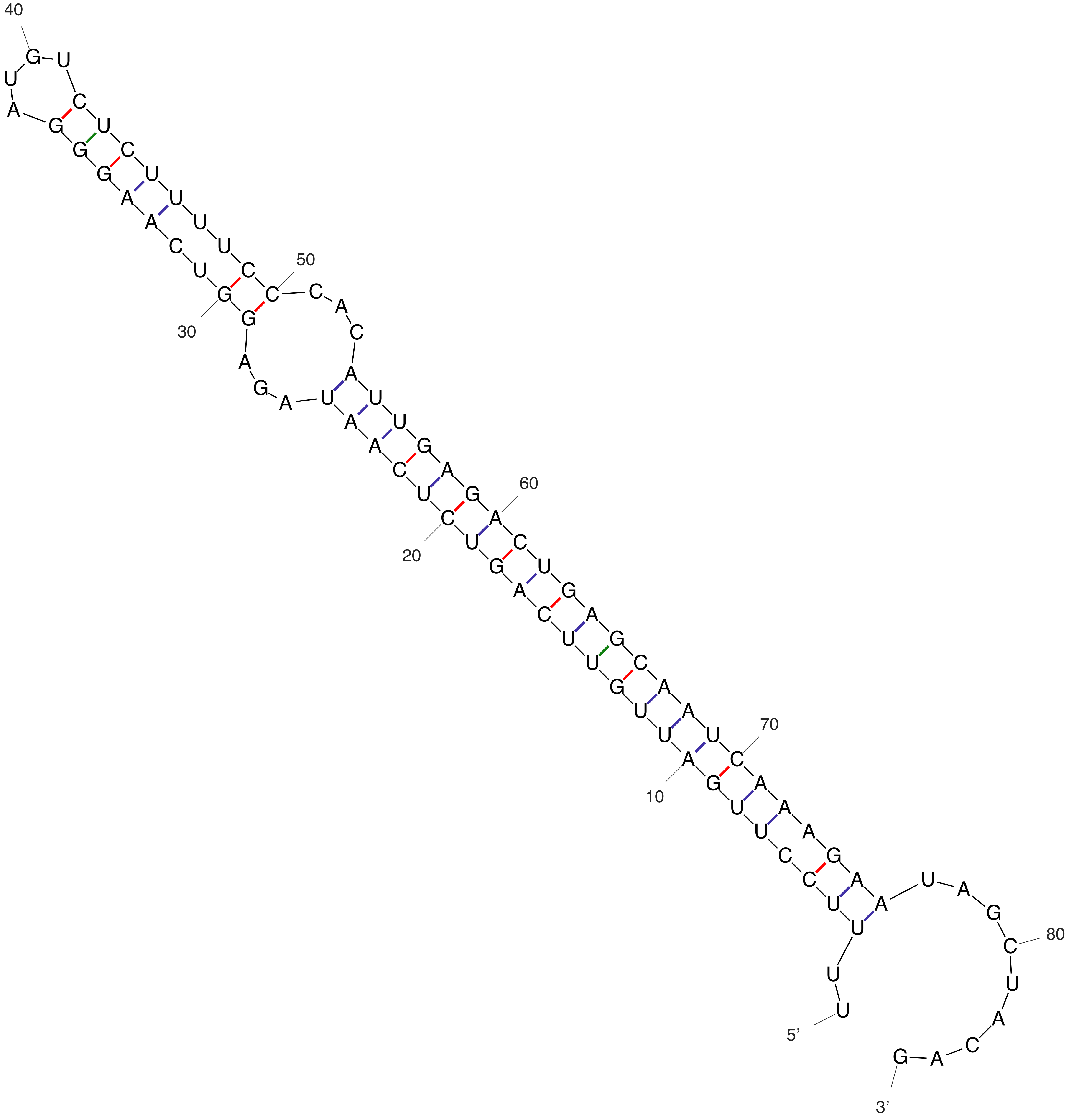


*dG = -37.50 [Initially -37.50] cme-miR5*

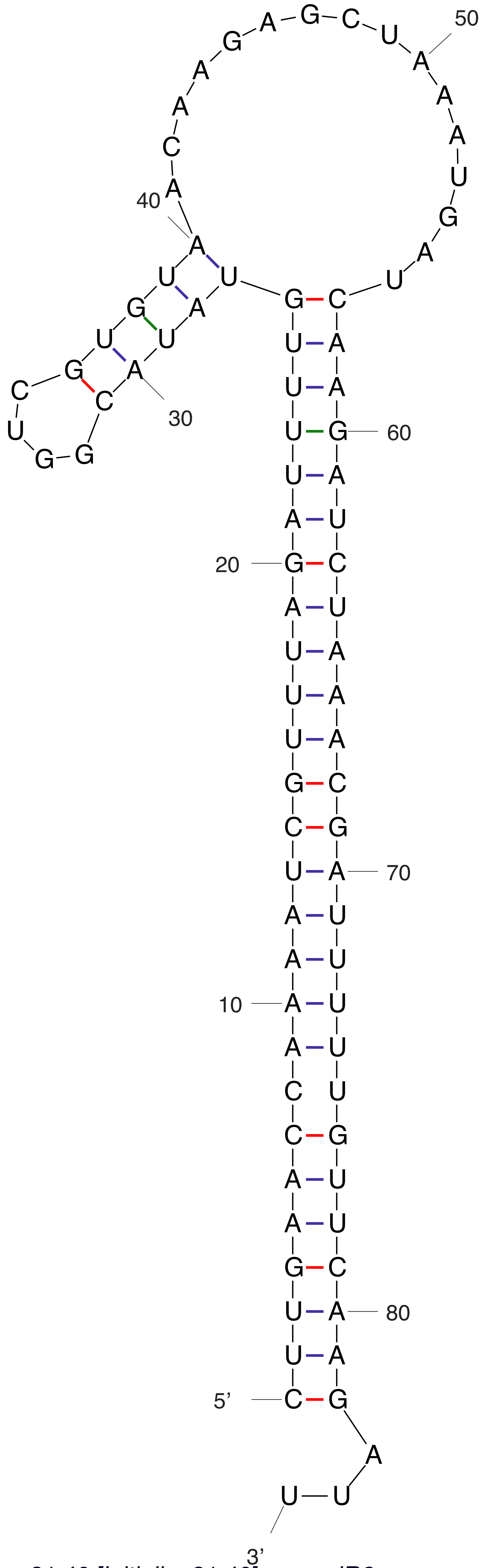


*dG = -31.40 [Initially -31.40] cme-miR6*

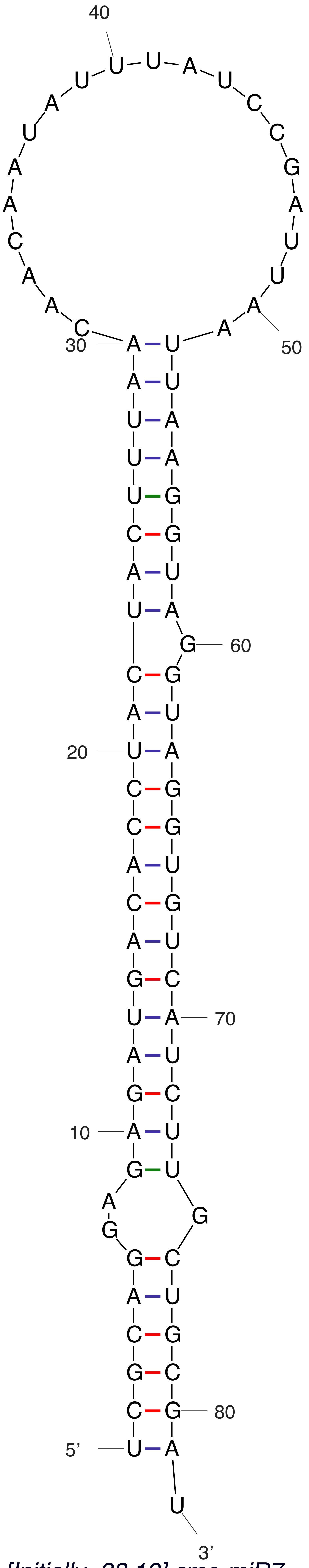


*dG = -38.10 [Initially -38.10] cme-miR7*

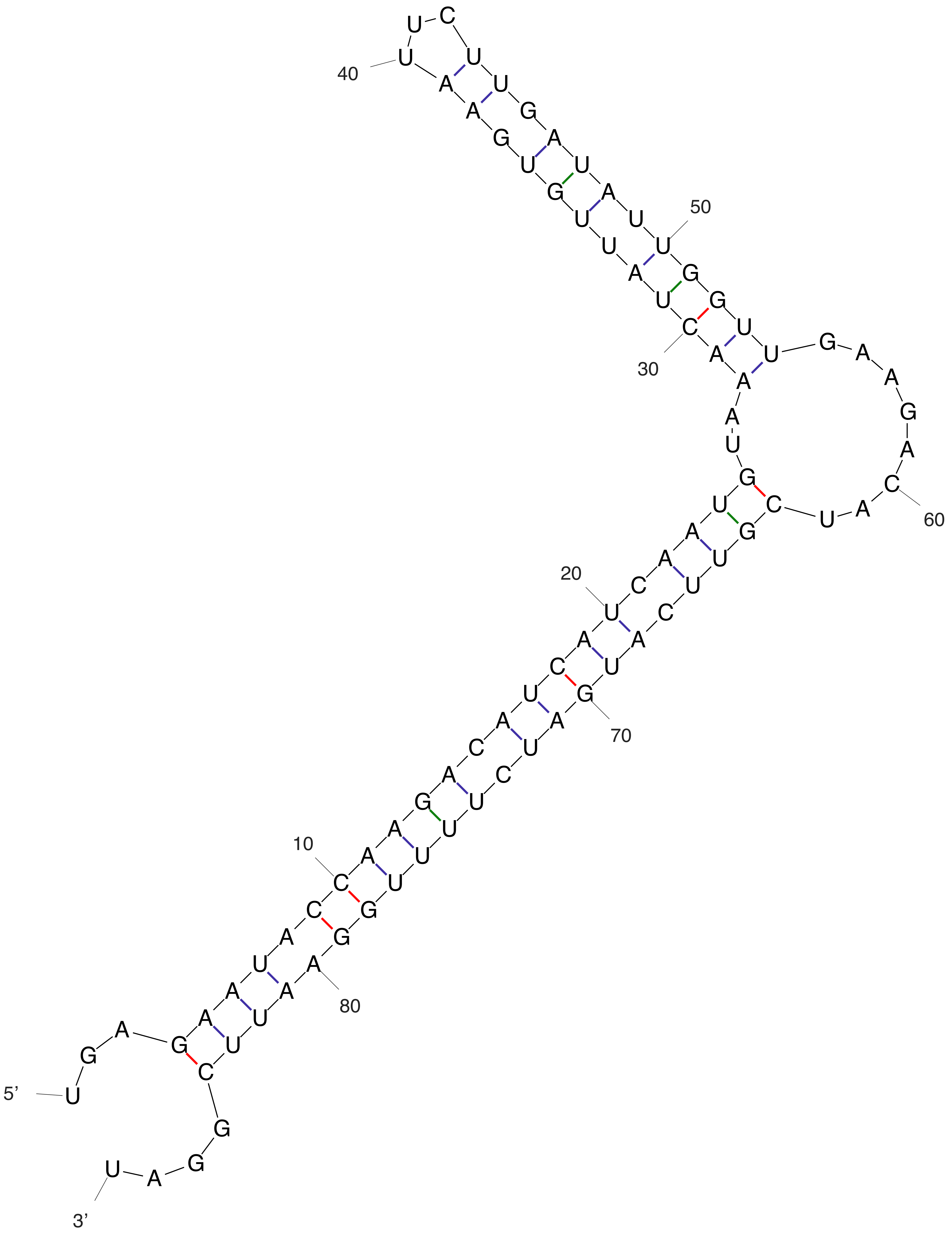


*dG = -18.50 [Initially -18.50] cme-miR8*

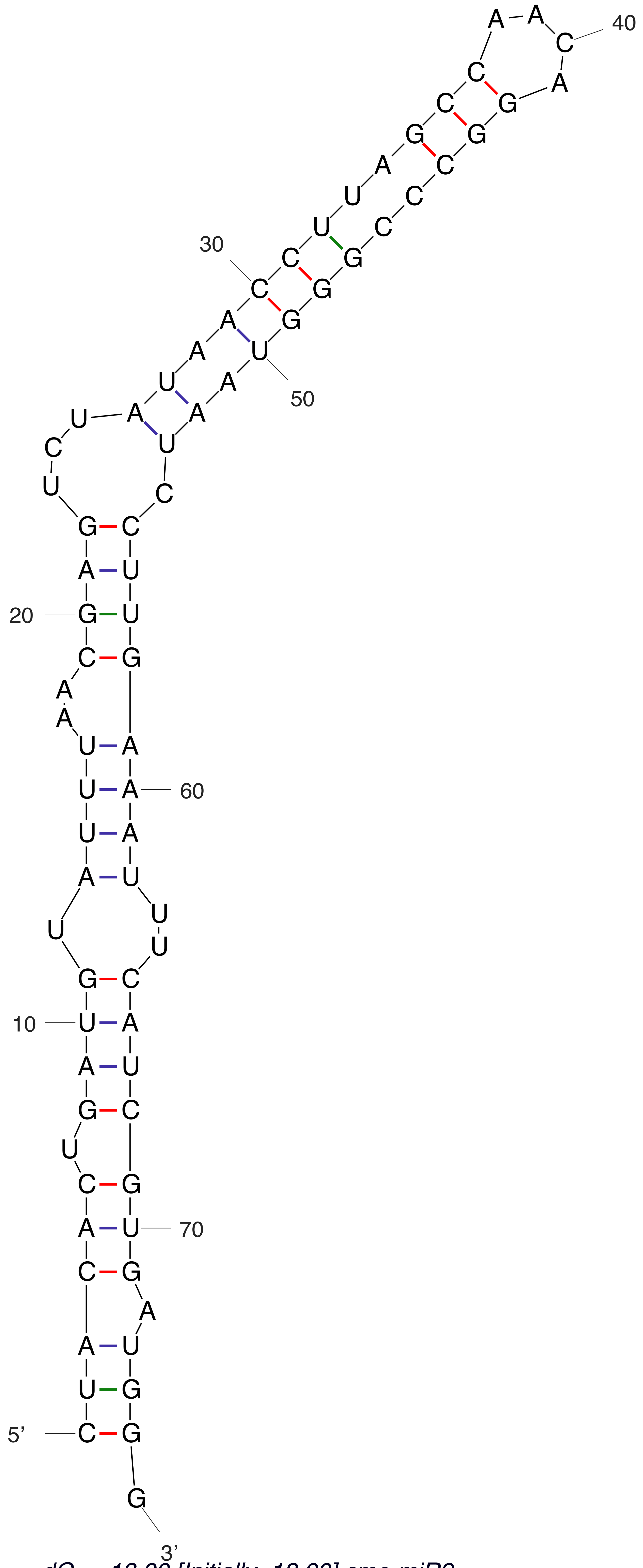


*dG = -18.00 [Initially -18.00] cme-miR9*
